# Coding Early Naturalists' Accounts into Long-Term Fish Community Changes in the Adriatic Sea (1800–2000)

**DOI:** 10.1371/journal.pone.0015502

**Published:** 2010-11-17

**Authors:** Tomaso Fortibuoni, Simone Libralato, Saša Raicevich, Otello Giovanardi, Cosimo Solidoro

**Affiliations:** 1 Department of Oceanography, Istituto Nazionale di Oceanografia e Geofisica Sperimentale, Sgonico, Italy; 2 Istituto Superiore per la Protezione e la Ricerca Ambientale, Chioggia, Italy; NIWA, New Zealand

## Abstract

The understanding of fish communities' changes over the past centuries has important implications for conservation policy and marine resource management. However, reconstructing these changes is difficult because information on marine communities before the second half of the 20^th^ century is, in most cases, anecdotal and merely qualitative. Therefore, historical qualitative records and modern quantitative data are not directly comparable, and their integration for long-term analyses is not straightforward. We developed a methodology that allows the coding of qualitative information provided by early naturalists into semi-quantitative information through an intercalibration with landing proportions. This approach allowed us to reconstruct and quantitatively analyze a 200-year-long time series of fish community structure indicators in the Northern Adriatic Sea (Mediterranean Sea). Our analysis provides evidence of long-term changes in fish community structure, including the decline of Chondrichthyes, large-sized and late-maturing species. This work highlights the importance of broadening the time-frame through which we look at marine ecosystem changes and provides a methodology to exploit, in a quantitative framework, historical qualitative sources. To the purpose, naturalists' eyewitness accounts proved to be useful for extending the analysis on fish community back in the past, well before the onset of field-based monitoring programs.

## Introduction

Natural fluctuations and human-induced modifications have caused long-term changes of marine fauna [1], [2]. The full appreciation of these changes and eventually their relation with driving forces, however, need a broadening of the time horizon through which we look quantitatively at ecosystem dynamics. Indeed, without a historical perspective, our perception of the marine environment might be consistently biased by knowledge of its recent status [3]–[5]. Therefore, the rescue and analysis of past records, which encompass literary, archival and scientific sources, for reconstructing a picture of what lived in the oceans in the past, is an important task [1], [6]–[9]. However, while historical quantitative data for some species may be available [5], information on marine communities before the second half of the 20^th^ century is, in most cases, anecdotal and merely qualitative [10], [11]. Thus, quantitative analysis of long-term changes at the community level, as well as integration of historical qualitative information with modern data, is not straightforward.

An objective intercalibration between qualitative and quantitative information, if possible, may add value to historical sources, allowing for integration of different types of data and reconstruction of long-term temporal trends of fish communities. This approach might be particularly important when analyzing the past century, during which both the dramatic acceleration of marine ecosystem degradation [6] and the transition from qualitative records to quantitative data occurred [9].

In the Mediterranean region the field-based monitoring programs for quantitative assessing the status of marine resources cover at most the last 30 years [12], failing to encompass the population dynamics of long-living species and the time scale of many natural and human-induced phenomena. However, other historical sources, at least for the past two centuries, might be locally abundant. In this context, the Northern Adriatic Sea (Mediterranean Sea) represents a valuable case-study due to the richness of both qualitative and quantitative historical sources on a large number of fish species, which allow performing the intercalibration between different kinds of information on fish communities. In particular, early naturalists' accounts of marine species were abundant since the beginning of the 19^th^ century, as a consequence of the ascendancy of the Linnaean system [13]. These documents, primarily based on observations of landings at fish markets and ports and on interviews with fishermen [14], typically consist of catalogues of species (Figure, 1) whose perceived abundance is described, along with insights into their main ecological features and notes on fishing gears and activities targeting them.

**Figure 1:**
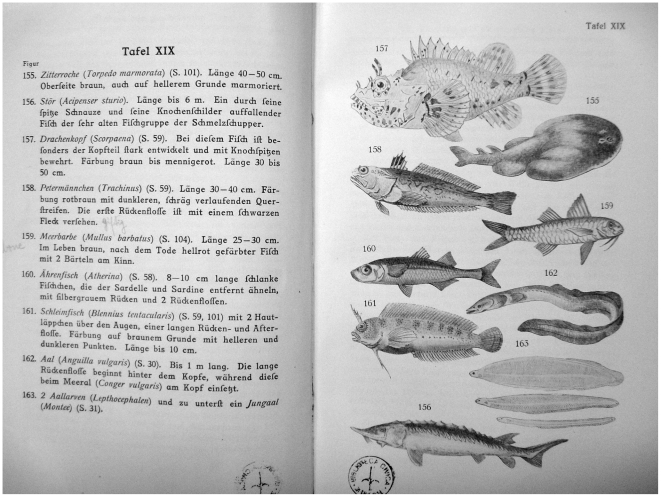
A naturalist's catalogue of species. Cori C.I. (Leipzig, 1910). Der naturfreund am strande der Adria und des mittelmeeregebietes.

We exploited these historical sources referred to the Northern Adriatic Sea to derive a coding of qualitative information provided by early naturalists into semi-quantitative one through an intercalibration with landing proportions. This allowed us to reconstruct a two centuries-long time series (1800–2000) of perceived abundance for many fish species. Long-term changes in the fish community structure were analyzed by applying a set of indicators based on *taxon*-specific properties. Results are discussed in the light of historical changes of fishery exploitation and other pressures in the basin.

## Materials and Methods

### Archival survey and data collection

We carried out an extensive survey of local archives, libraries and museums in Venice, Padua, Rome, Trieste, Chioggia (Italy) and Split (Croatia) to collect naturalists' descriptions of Adriatic marine fauna (first dataset) and landing statistics (second dataset) from the beginning of the 19^th^ century onwards.

The first dataset contained information on fish species reported in 36 naturalists' books published between 1818 and 1956 (Table S1). We updated species synonymies according to modern nomenclature, and species' lists were checked for accuracy, resulting in the description of 255 fish species. The perceived abundance of these species, recorded in each naturalists' account, was ranked using a four-level class coding system (i.e., very rare, rare, common and very common). However, some information might not have been independent because some authors based their reports on former naturalists' accounts. Therefore, to minimize this source of bias and to overcome problems associated with missing data and non-homogeneity of sampling, we aggregated observations by taking the modal perceived abundance value of each species over 25-year-long periods from 1800 to 1950 (Materials and Methods S1).

The second dataset consisted of landing statistics from major fish markets or wide coastal areas; these data covered the period between 1874 and 2000 (Table S2). Annual landing data were available for approximately 100 species/groups of species (Table S3) in terms of wet weight (kg/year). The taxonomic resolution of landing statistics was not homogeneous among different sources and periods. Major differences regarded the taxonomic class Chondrichthyes. Few sources, in fact, distinguished between different species of sharks or rays, while in most cases they were classified in wide categories according to their size and morphology. In order to take into account for this issue the intercalibration and integration of naturalists' perceived abundance and landings was performed by transforming data into groups with homogeneous taxonomic resolution. We used these data to estimate the average proportion of each species in the landings for each 25-year-long period (Materials and Methods S1). Being biased towards commercial species and not standardized in terms of fishing effort or fishing gear, landings have the intrinsic limitations of fishery-dependent data for quantitative single species assessment. However, landing proportions (i.e., observed relative composition, Materials and Methods S1) are useful because they indicate changes in the composition of exploited fish communities [9], [15].

### Intercalibration and integration of qualitative and quantitative data

**Figure 2:**
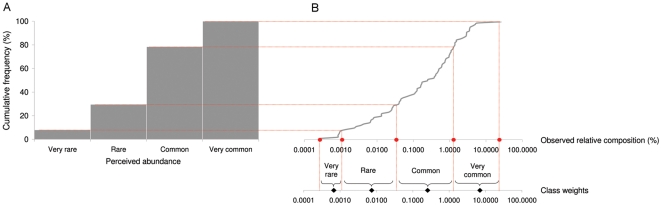
Intercalibration between qualitative (perceived abundance) and quantitative (observed relative composition) data in the period 1901–1925. (a) Cumulative frequency distribution of the number of species against qualitative classes of perceived abundance; (b) cumulative frequency distribution of the number of species against quantitative observed relative composition. Red dots represent the class limits that subdivide the species into four groups whose cumulative frequency distribution is the same of the frequency distribution of naturalists' classes of perceived abundance. Black dots represent the class weights associated to each class of perceived abundance.

We used periods with overlapping information (1876–1900, 1901–1925 and 1926–1950; N = 98 species/groups of species) for the intercalibration between the two datasets (Materials and Methods S1). For each period a comparison of the cumulative frequency distributions of the number of species by qualitative classes of perceived abundance (Figure 2a) and the number of species by quantitative observed relative composition (Figure 2b) permitted the following: *i)* the identification of thresholds (i.e., class limits) corresponding to percentile values that discriminate among qualitative classes (Table S4), thus allowing *ii)* the mapping of quantitative landing data for the periods 1951–1975 and 1976–2000 into qualitative classes of perceived abundance and *iii)* the ability to assign a numerical weight (i.e., class weight) to each qualitative class as the geometric mean of the class limits (Figure 2b) (Table S5). We used a jack-knife resampling technique [16] to estimate the median, mean and percentiles for the class limits and class weights by replicating the entire estimation procedure 1000 times for the three periods with overlapping information. For each replicate, 10 randomly extracted data were excluded from both datasets.

The integration of the two datasets and the reconstruction of the 200-year-long time series of fish perceived abundance was done by transforming the landing data for the periods 1951–1975 and 1976–2000 into classes of perceived abundance by using the median values of the whole set of 3000 class limit replicates (i.e., global class limits, Table S4). We verified the acceptability of this choice by testing the null hypothesis that errors made in reconstructing species' perceived abundance using median values of class limits were equal to errors made using a random classification (Materials and Methods S1).

### Fish community structure indicators

We calculated the fish community structure indicators (*Z_p_*) for each period (*p*) as the weighted averages of time-independent *taxon*-specific properties (*z_i_*), i.e., trophic level, taxonomic group, functional group, maximum body length and age at sexual maturity (Table S6), each grouped into sub-categories (Table S7) [17]. The indicators, representing weighted proportions of groups of species in the community, were calculated as:
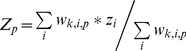
(1)where *w_k,i,p_* is the class weight for *taxon i* belonging to the class of perceived abundance *X_k_* in the period *p*. We computed community structure indicators using the whole set of 3000 class weight replicates obtained from the jack-knife resampling procedure.

We performed the analysis of temporal trends using a linear regression of the median values of the fish community structure indicators, where we considered *α* = 0.1 as an appropriate threshold of significance for these inherently noisy data. While the naturalists' dataset contained information for 255 species, landing statistics for the periods 1951–1975 and 1976–2000 reported information for 87 species/groups of species. Accordingly, we performed the analysis on *i)* the entire naturalists' dataset (N = 255 species) for the period 1800–1950 and *ii)* the subset comprising the 87 species/groups of species for the period 1800–2000.

## Results

**Figure 3:**
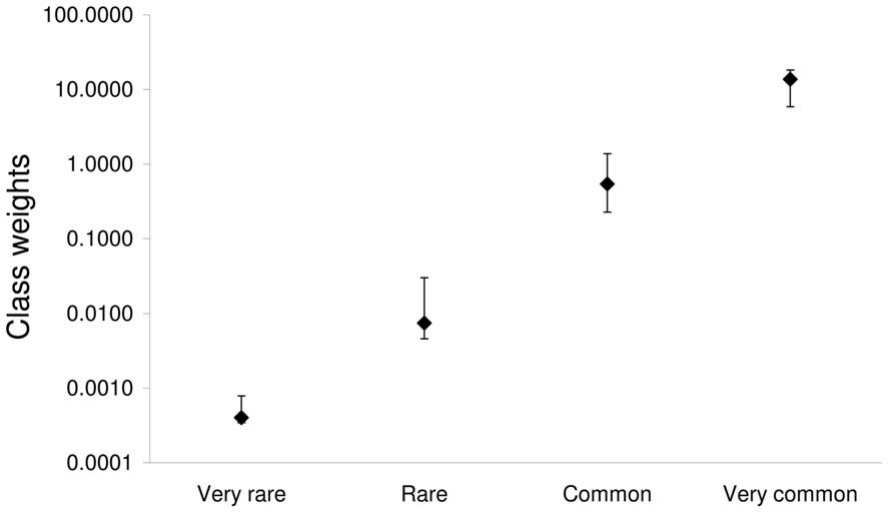
Class weights resulting from the intercalibration of qualitative and quantitative data. The median and the interquartile range (N = 3000) are reported (jack-knife resampling technique). The Y-axis is in a logarithmic scale.

Sets of class limits and weights resulting from the intercalibration were slightly different in the three periods with overlapping information (1876–1900, 1901–1925 and 1926–1950; Table S4). However, they all followed a logarithmic scale (Figure 3).

The comparison between classes of perceived abundance reconstructed using global class limits (median values) and those observed by naturalists (for the periods with overlapping information) showed agreement at *p* = 10^−6^ (see supporting information). This test supported the use of this coding system to reconstruct fish perceived abundances for the periods 1950–1975 and 1976–2000 on the basis of observed relative composition derived from landings, thus increasing the reliability of the reconstructed 200-year-long time series of perceived abundances of 87 species/groups of species. Moreover, this corroboration of the global class limits supported the use of median values for class weights for analyzing fish community structure indicators trends over time.

The analysis of long-term temporal trends of indicators highlighted changes in the fish community structure, both considering the entire naturalists' dataset (N = 255 species) for the period 1800–1950 (Table S8) and the subset of species (N = 87 species/groups of species) for the period 1800–2000 (Table S9).

### Naturalists' descriptions: 1800–1950, 255 species

**Figure 4:**
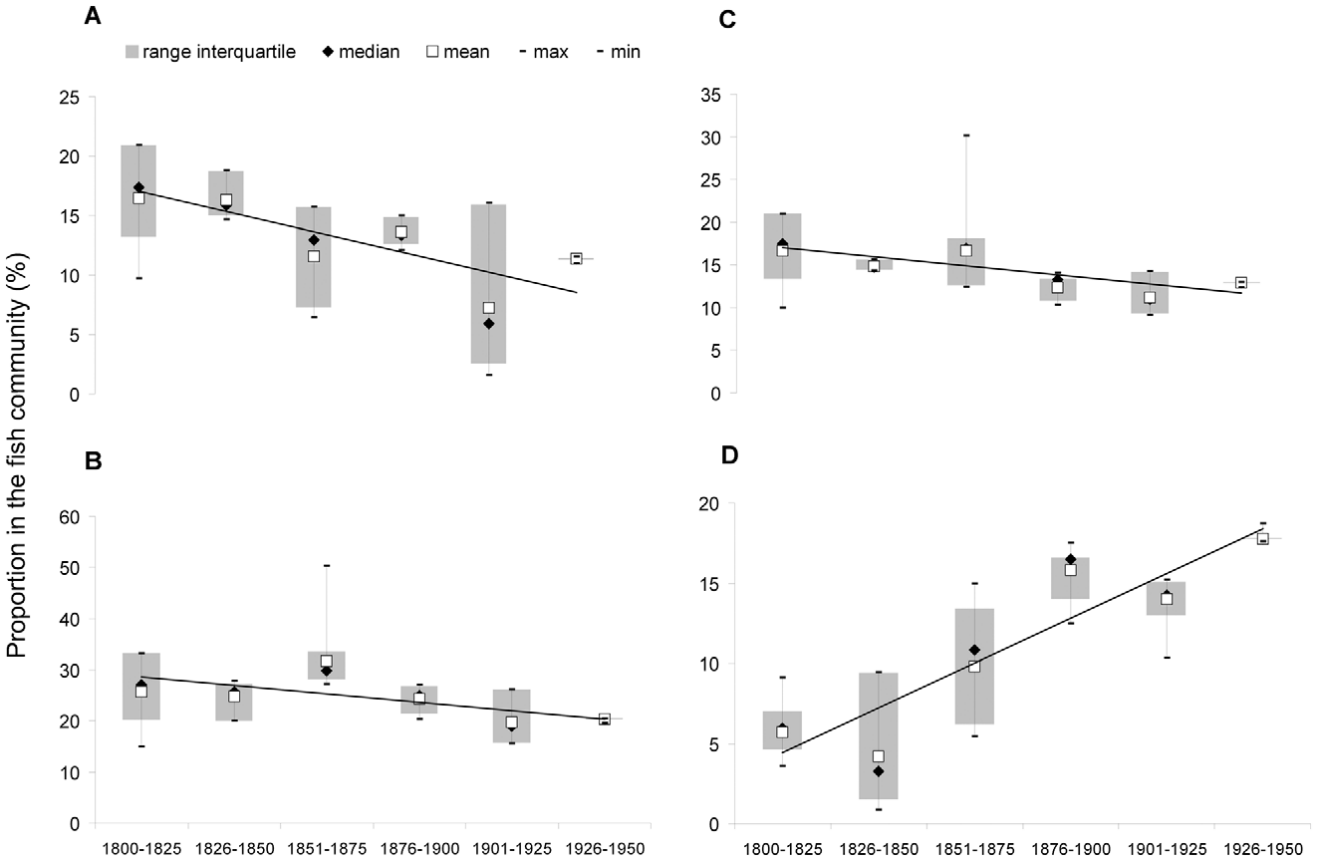
Temporal trends of fish community structure indicators in the period 1800–1950 (N = 255 species). (a) Chondrichthyes; (b) large demersals; (c) species with a maximum body length between 120 and 250 cm; (d) species that reach sexual maturity within the 1^st^ year of life.

A significant decline in the proportion of Chondrichthyes in the fish community from 17.3% to 11.4% was observed (*β* = −1.692, *r^2^* = 0.627, *p* = 0.06; Figure 4a). Large demersals proportion significantly declined from 27% to 20.4% (*β* = −1.671, *r^2^* = 0.577, *p* = 0.079; Figure 4b) and large-sized species (maximum body length between 120 and 250 cm) significantly declined from 17.5% to 13% (*β* = −1.066, *r^2^* = 0.65, *p* = 0.053; Figure 4c). Conversely, the proportion of small-sized species (maximum body length between 25 and 55 cm) and fast-maturing species (species that reach sexual maturity within the 1^st^ year of life) in the community significantly increased from 11.7% to 28.2% (*β* = 4.276, *r^2^* = 0.779, *p* = 0.02), and from 5.9% to 17.8% (*β* = 2.788, *r^2^* = 0.799, *p* = 0.016; Figure 4d), respectively. The mean trophic level substantially did not change (Table S8).

### Integration between naturalists' descriptions and landings: 1800–2000, 87 species/groups of species

**Figure 5:**
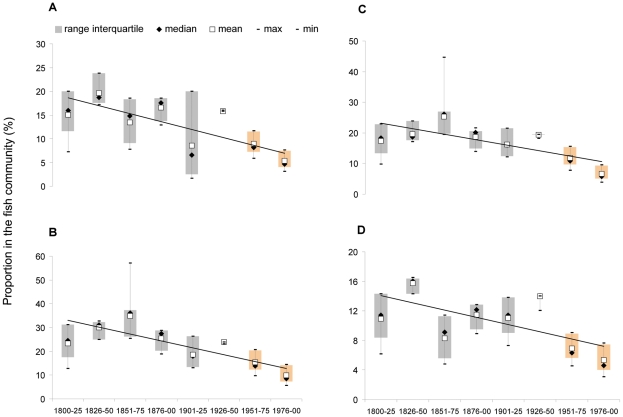
Temporal trends of fish community structure indicators in the period 1800–2000 (N = 87 species/groups of species). Grey boxes indicate the naturalists' observations of species' perceived abundance, and orange boxes indicate the observed relative composition transformed into classes of perceived abundance. (a) Chondrichthyes; (b) large demersals; (c) species with a maximum body length between 120 and 250 cm; (d) species that reach sexual maturity between the 4^th^ and 6^th^ years of life.

A significant decline in the proportion of Chondrichthyes in the fish community from 15.9% to 4.6% was observed (*β* = −1.664, *r^2^* = 0.548, *p* = 0.036; Figure 5a). Significant declines were also observed for the proportion in the fish community of large demersals from 24.4% to 8.5% (*β* = −2.898, *r^2^* = 0.611, *p* = 0.022; Figure 5b), mid-sized species (maximum body length between 55 and 120 cm) from 31.8% to 17.3% (*β* = −3.901, *r^2^* = 0.517, *p* = 0.044), large-sized species (maximum body length between 120 and 250 cm) from 18.3% to 5.8% (*β* = −1.793, *r^2^* = 0.506, *p* = 0.048; Figure 5c) and late-maturing species (species that reach sexual maturity between 4 and 6 years of life) from 11.4% to 4.6% (*β* = −0.979, *r^2^* = 0.398, *p* = 0.093; Figure 5d). The mean trophic level declined from 3.56 to 3.16, even though this decline was not significant (*p* = 0.155, Table S9).

## Discussion

In the last years a growing number of papers in fisheries and marine ecology demonstrated that focusing too narrowly on recent decades, not taking fully into account historical records, could lead to the so called “shifting baseline syndrome” [3]. This implies that severe depletions of resources with respect to pristine state can be not recognized due to the “historical myopia” of fishery scientists [11], [18]–[21]. However, while quantitative past information to reconstruct pristine densities could be available for few commercially important species (e.g., Rosenberg et al. 2005), analyses at the community level are more difficult to carry out because quantitative data referred to many *taxa* are usually not available over long time-frames.

In this work we collected historical information on the perceived abundance of 255 fish species and we implemented a novel approach to intercalibrate qualitative and quantitative data. The coding method allowed the integration of different sources of information, thus providing the basis for the analysis of changes in fish community structure over the past two centuries in the Northern Adriatic Sea. The decline of Chondrichthyes, large demersals and large-sized species is the most notable signal we detected in this study since it was observed both analyzing the whole community (255 species) for the 1800–1950 time frame (naturalists' dataset) and the subset of commercial species (87 species/groups of species) over 2 centuries (1800–2000, integrated dataset) (Figures 4 and 5; Tables S8 and S9).

Although relevant methodological differences (e.g., coding system, metric, time frame, number of species, indicators) hamper the direct quantitative comparison, the decline of Chondrichthyes, large demersals, large-sized and late-maturing species observed in our study is in accordance with previous studies referred to the Adriatic and the Mediterranean Sea [6], [22]. Indeed, Chondrichthyes are highly vulnerable to anthropogenic disturbances, and especially to fishery, because of their life-history traits (low fecundity, slow growth, large size and late maturity) [23], [24], and warnings of their depletion in the Adriatic Sea had already sounded in the 20^th^ century [25], [26]. Similar considerations on the vulnerability to fishery apply for large demersals (e.g., the European hake, *Merluccius merluccius*, and the angler, *Lophius piscatorius*), large-sized and late-maturing species (e.g., the dusky grouper, *Epinephelus marginatus*, and the brill, *Scophthalmus rhombus*) [27]. Nevertheless, the long-term quantitative analysis of a large number of species presented here revealed also other significant changes in the community structure, such as the increase of small-sized and early-maturing species in the 1800–1950 period (Figure 4d and Table S8).

Disentangling the role of main driving forces in shaping all these long-term community changes is, however, far from trivial. Indeed, the Northern Adriatic Sea has historically been subjected to several anthropogenic sources of ecological disturbance and natural fluctuations, including eutrophication, water pollution, species invasion, habitat alteration, climatic changes and fishing activities [6], [28]–[30], all of which potentially affected fish community structure and composition.

Nutrient enrichment in the basin, for instance, began in 1900, and human-induced coastal and estuarine habitat alteration has occurred at least since the 17^th^ century and even before that on the northwestern Adriatic coast [6]. However, critical signs of anthropogenic eutrophication (e.g., benthic anoxias, dystrophic events) were first detected only in the second half of the 20^th^ century [28] and habitat alteration has dramatically intensified after 1950 [30]. Similarly, water pollution and species invasion mainly occurred in the last half of the 20^th^ century [30]. Fishery in the Northern Adriatic Sea has been characterized by a tradition of intense exploitation that dates back, at least, to the begin of the 19^th^ century [29]. However, the fishing activities in this basin became industrialized in the second half of the 20^th^ century, with the introduction of the engine propeller, new and more effective fishing gears (e.g., iron-teethed dredges, mid-water pelagic trawls) and technologies (e.g., freezer trawlers, radar and echo sounding).

In the last half of the 20^th^ century, therefore, all these driving forces had heavy impacts on the ecosystem and their effects are difficult to disentangle and might necessarily be considered together for explaining the observed community changes. Before 1950, however, fishing pressure might have played a more important role. The decline of Chondrichthyes and top predators proportion over the two centuries are consistent with this hypothesis. It is worth noting that the decline of Chondrichthyes, large demersals and large-sized species was confirmed both analyzing the whole fish community (1800–1950, 255 species) and the subset of commercial species (1800–2000, 87 species/groups of species), indicating that observed trends are not merely a consequence of changes in target species, taxonomic resolution of landing statistics and improvement in fishing technology due to fishery industrialization.

In principle the increase of small-sized/early-maturing species proportion in the fish community from 1800 to 1950 might be related to the slight increase in eutrophication occurred in the basin [28]. However, this process would have favoured also upper trophic levels, due to bottom-up cascading effects [31]. On the contrary, our findings, by showing a decline in the proportion of top predators and large-sized species in the community together with the lack of any significant increase in the proportion of small pelagics (mainly planktivorous species), suggest a limited response of the fish community to this source of disturbance. Conversely, the observed trends are compatible with a top-down effect driven by fishing activities: the significant decline of Chondrichthyes, top predators and late-maturing species proportion in the community are consistent with effects induced by fishing, and the increase in small-sized/early-maturing species might have been driven by a predation release, fostered by the removal of top predators [31]. Long term effects of fishing are also in accordance with the evidence that, already by the end of the 19^th^ century, fishery was considered responsible for stock depletion [32] and, few decades later, fishery was proved to be responsible for the structural changes in fish communities in the area [25].

Moreover, the decline of top-predators and late-maturing species and the increase of small-sized species proportion in the community are compatible with a long-term “fishing down the food web” effect [15]. Although the decrease in the mean trophic level between 1800 and 2000 was not significant, the rate of mean trophic level decline quantified here is probably underestimated due to taxonomic over-aggregation and neglection of species ontogenic shift in trophic level quantification, that tend to mask the “fishing down” process [33]. Furthermore, in the present analysis only fish were considered, whereas the observed trend of the mean trophic level might have been more marked if invertebrates were included in the analysis [15], [34].

Results globally suggest a framework consistent with a top-down control predominating on a bottom-up one before 1950, and support the hypothesis that the effects of fishing prevailed on other sources of ecological disturbance. Furthermore, results indicate that pre-industrial fisheries had already had significant impacts on the Northern Adriatic fish community.

Naturalists' eyewitness accounts of fish species, which have long been disregarded by fishery biologists as being “anecdotal” and not “science” [35], proved to be a useful tool for extending the analysis into the past, well before the onset of field-based monitoring programs. It is worth noting that the coding of naturalists' accounts might also be useful in establishing historical baselines for threatened species. In the case of the Adriatic Sea, for example, these sources revealed that some species, such as the angel shark, *Squatina squatina*, the tope shark, *Galeorhinus galeus*, and the sturgeon, *Acipenser sturio*, which are now considered extirpated [24], were common until 1950. Our methodology could contribute to assessing the magnitude of long-term change in proportion of a species in the community and to addressing the conservation status of species whose assessment is not straightforward because of a lack of quantitative data.

The evidence that class weights follow a logarithmic scale and the result that temporal trends of fish community structure indicators are not very sensitive to changes in the logarithm base (Materials and Methods S1, Figures S1 and S2), suggest that log metrics should be used when dealing with qualitative descriptions of species perceived abundance. This result is of particular interest since it provides further evidence to support the conclusion from cognitive studies that logarithmic metrics are dominant when numbers are so large that accurate counting is not practical [36], as is the case for naturalists' observations at fish markets and ports. Moreover, this finding does not support the choice of linear-based classification systems used in previous historical ecology studies [6], [37].

The method proposed here and the metric we derived could be applied to other case studies where qualitative and quantitative information need to be combined, allowing for the extraction of information from old—and somehow overlooked—sources and enable a rediscovery of the importance of testimonies from earlier naturalists, fishermen, travelers and historians [3], [7], [10], [20], [38].

## Supporting Information

Figure S1 Sets of class weights (N = 250) used to test the sensitivity of temporal trends of fish community structure indicators to the logarithmic base. The set of class weights derived from the intercalibration (estimated base = 35.1) is represented by white squares. (TIF)

Figure S2 Graphs showing how *β* and the *P*-value of temporal trends of fish community structure indicators vary when using different sets of class weights. *(a)* large demersals; *(b)* Chondrichthyes; *(c)* species that reach sexual maturity between the 4^th^ and 6^th^ years of life; *(d)* species with a maximum body length between 120 and 250 cm. (TIF)

Table S1 List of naturalists' books that were analyzed. (DOC)

Table S2 Sources of landing data. (DOC)

Table S3 Taxonomic groups for which landing statistics were available for the periods 1876–1900, 1901–1925 and 1926–1950. (DOC)

Table S4 Class limits (median and interquartile range) for each period of intercalibration and for the entire period with overlapping information that discriminate the qualitative classes of perceived abundance. (DOC)

Table S5 Class weights, computed using the intercalibration, and their association with classes of perceived abundance. (DOC)

Table S6 List of species described in naturalists' documents and the species' ecological characteristics according to Fishbase. (DOC)

Table S7 Categories of *taxon*-specific properties based on information from Fishbase. (DOC)

Table S8 Results of the analysis of trends for fish community structure indicators (N = 255 species; years 1800–1950), where significant (*α* = 0.1) slopes are shown in bold. (DOC)

Table S9 Results of the analysis of trends for fish community structure indicators (N = 87 species/groups of species; years = 1800–2000), where significant (*α* = 0.1) slopes are shown in bold. (DOC)

Materials and Methods S1 (DOC)
