# Supplementary figures and images for: Coding Early Naturalists' Accounts into Long-Term Fish Community Changes in the Adriatic Sea (1800–2000)
Source: PLoS One. 2010 Nov 17;5(11):e15502. doi: 10.1371/journal.pone.0015502 (PMC2984504; doi:10.1371/journal.pone.0015502)

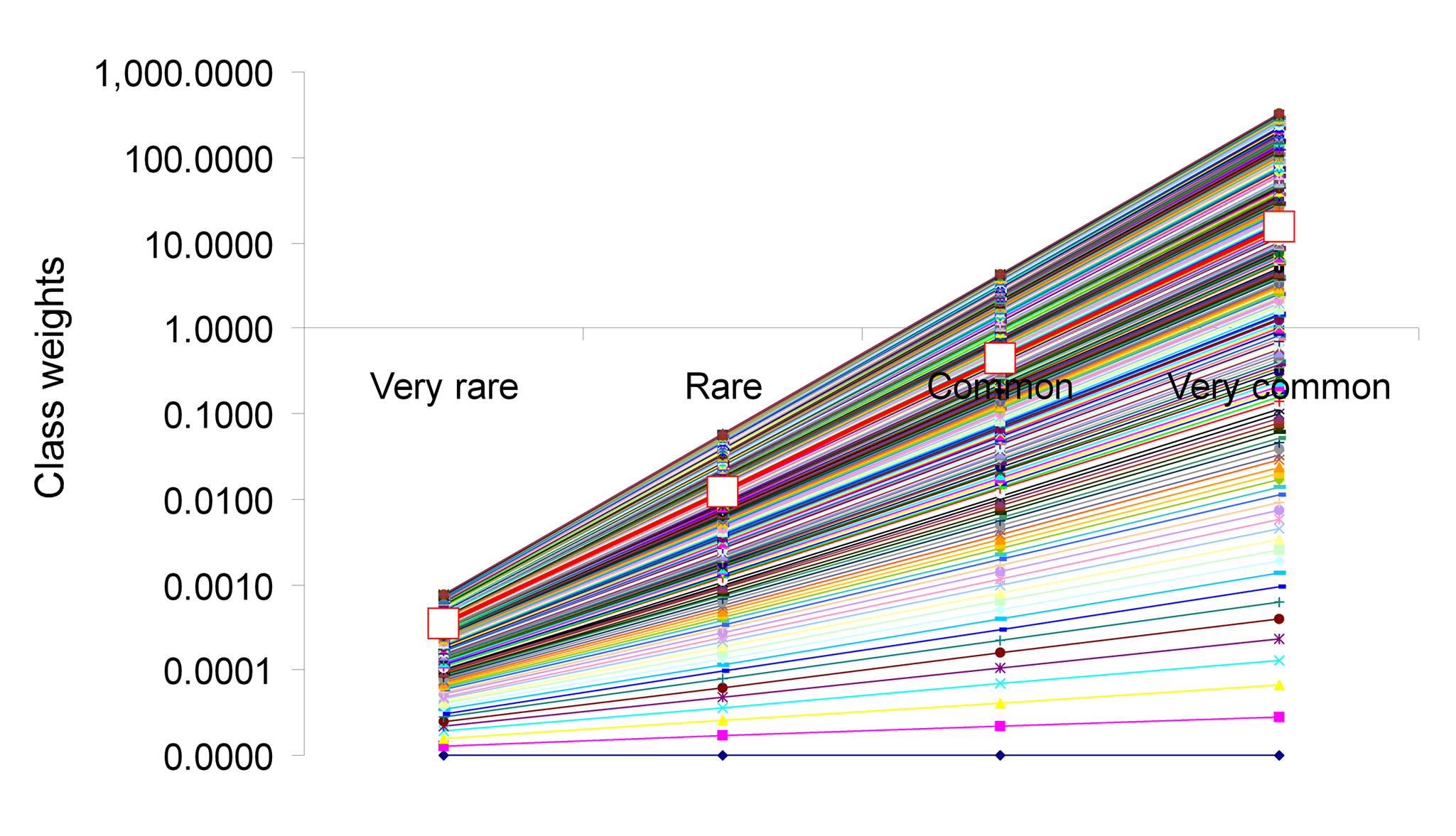

Supplement: Figure S1 — Sets of class weights (N = 250) used to test the sensitivity of temporal trends of fish community structure indicators to the logarithmic base. The set of class weights derived from the intercalibration (estimated base = 35.1) is represented by white squares. (TIF) [file pone.0015502.s001.tif]

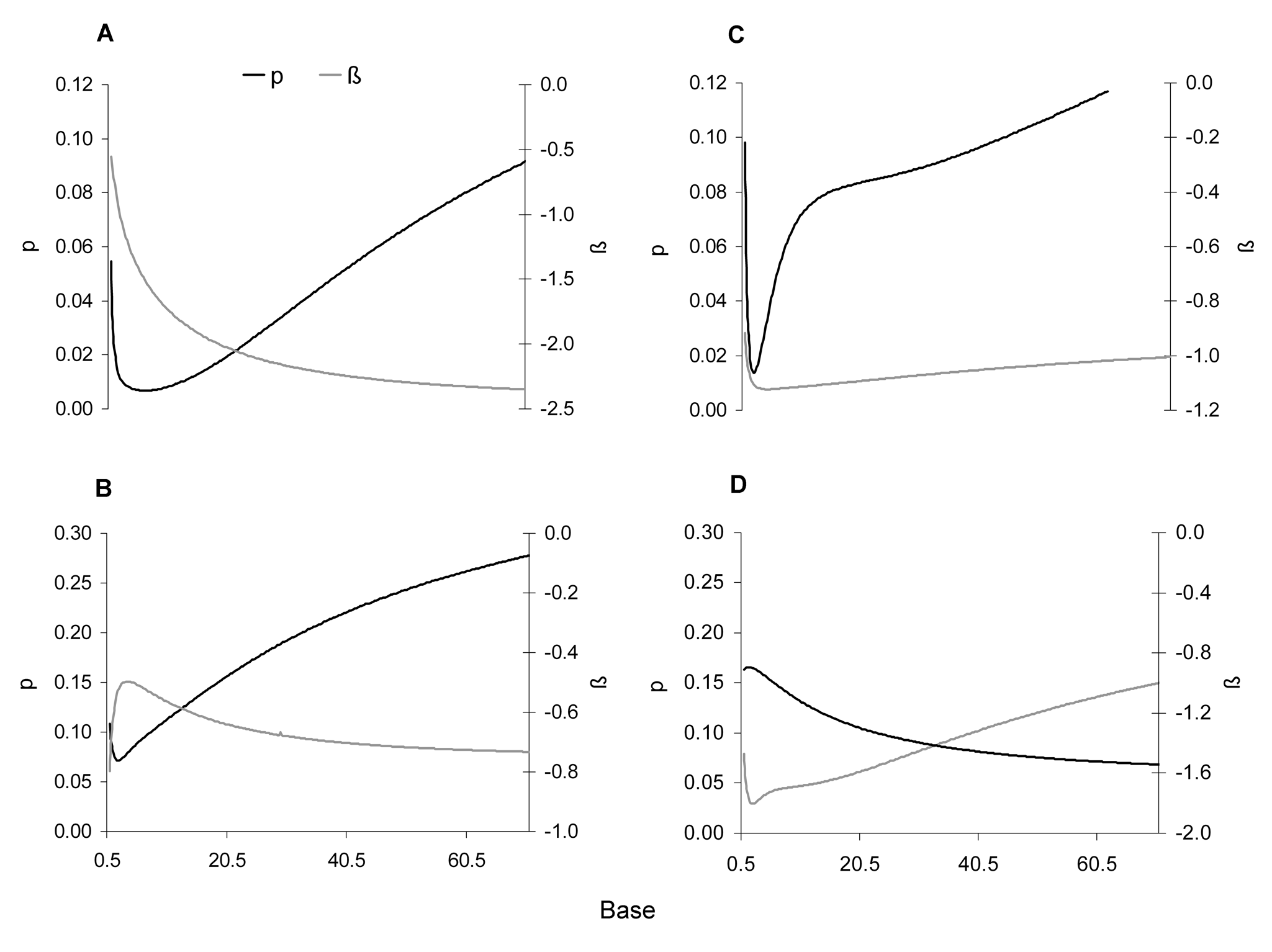

Supplement: Figure S2 — Graphs showing how β and the P-value of temporal trends of fish community structure indicators vary when using different sets of class weights. (a) large demersals; (b) Chondrichthyes; (c) species that reach sexual maturity between the 4th and 6th years of life; (d) species with a maximum body length between 120 and 250 cm. (TIF) [file pone.0015502.s002.tif]
