# Supplementary material for: Coding Early Naturalists' Accounts into Long-Term Fish Community Changes in the Adriatic Sea (1800–2000)
Source: PLoS One. 2010 Nov 17;5(11):e15502. doi: 10.1371/journal.pone.0015502 (PMC2984504; doi:10.1371/journal.pone.0015502)
Supplement: Table S1 — List of naturalists' books that were analyzed. (DOC) [file pone.0015502.s003.doc]

Table S1. List of naturalists’ books that were analyzed.

| Year | Place | Author | Title | N° of species described | N° of valid species |
| --- | --- | --- | --- | --- | --- |
| 1818 | Treviso* | S. Chiereghin | Descrizione de' pesci, de' crostacei, e de' testacei che abitano le lagune ed il Golfo veneto | 139 | 120 |
| 1822 | Pavia | F. L. Naccari | Ittiologia Adriatica ossia catalogo de' pesci del Golfo e lagune di Venezia | 118 | 90 |
| 1823-24 | Chioggia | G. D. Nardo | Descrizione di un pesce raro dell'Adriatico, ed osservazioni ittiologiche dedicate al signor Giuseppe Cernazai da G. Domenico Nardo di Chioggia | 162 | 120 |
| 1824 | Ulm | G. V. Martens | Reise nach Venedig | 144 | 111 |
| 1827 | Pavia | G. D. Nardo | Prodromus observationum et disquisitionum Adriaticae ichthyologiae | 156 | 129 |
| 1832-41 | Rome | C.L. Bonaparte | Iconografia della fauna italica per le quattro classi degli animali vertebrati - tomo III (pesci) | 58 | 54 |
| 1846 | Trieste | E. Plucar | Der Fischplatz zu Triest, oder Aufzählung und populäre Beschreibung der demselben aus dem adriatischen Golfe zugeführten Fische und anderen essbaren Meerproduckte nebst Andeutung ihrer Zubereitung als Speise | 118 | 109 |
| 1847 | Venice | G. D. Nardo | Prospetto della fauna marina volgare del Veneto estuario con cenni sulle principali specie commestibili dell'Adriatico | 168 | 123 |
| 1860 | Venice | G. D. Nardo | Prospetti sistematici degli animali delle Province venete e del Mare Adriatico e distinzione delle specie in gruppi relativi alla loro geografia fisica ed all'interesse economico statistico che presentano | 306 | 218 |
| 1866 | Trieste | A. Perugia | Catalogo dei pesci dell'Adriatico | 265 | 223 |
| 1869 | Trieste | A. Stossich | Elenco sistematico degli animali del Mare Adriatico riuniti nella separata divisione della fauna adriatica del Museo | 228 | 195 |
| 1870 | Venice | A. P. Ninni | Enumerazione dei pesci delle Lagune e Golfo di Venezia con note | 249 | 220 |
| 1872 | Genoa | A. Targioni Tozzetti | La pesca in Italia | 255 | 212 |
| 1874 | Milan | G. Canestrini | Fauna d'Italia - parte terza (pesci) | 245 | 216 |
| 1875 | Venice | E. F. Trois | Prospetto sistematico dei pesci dell'Adriatico e catalogo della collezione ittiologica del R. Istituto Veneto | 228 | 204 |
| 1876 | Trieste | S. De Syrski | Relazione sulle osservazioni fatte in seguito a disposizione dell'I.R. Governo Marittimo riguardo al tempo della frega degli animali esistenti nel Mare Adriatico | 96 | 96 |
| 1879 | Trieste | M. Stossich | Prospetto della fauna del mare Adriatico - parte I (pesci) | 256 | 227 |
| 1879-80 | Palermo | P. Doderlein | Manuale ittiologico del Mediterraneo, ossia Sinossi metodica delle varie specie di pesci riscontrate sin qui nel Mediterraneo ed in particolare nei mari di Sicilia | 54 | 46 |
| 1880 | Venice | A. P. Ninni | Saggio dei prodotti acquatici e dell'industria peschereccia delle lagune e mare di Venezia | 194 | 179 |
| 1881 | Trieste | A. Perugia | Elenco dei pesci dell'Adriatico | 235 | 208 |
| 1880-81 | Venice | M. L. Sormani | La Provincia di Venezia: monografia statistica, economica, amministrativa raccolta e coordinata dal conte Luigi Sormani Moretti regio prefetto | 312 | 240 |
| 1882 | Trieste | C. De Marchesetti | La pesca lungo le coste orientali dell'Adria | 195 | 177 |
| 1881-82 | Split | J. Kolombatovic | Mammiferi, anfibi e rettili della Dalmazia e pesci rari e nuovi per l'Adriatico che furono catturati nelle acque di Spalato | 70 | 61 |
| 1883 | London | G. L. Faber | The fisheries of the Adriatic and the fish thereof | 376 | 252 |
| 1891 | Wien | S. Brusina | Due elenchi dei pesci della Dalmazia di M. Botteri coll'aggiunta di Heckel, Bellotti, Stalio ecc. | 259 | 246 |
| 1895 | Trieste | V. L. Sucker | Die Fische nebst den essbaren wirbellosen Thieren der Adria und ihre Zubereitung | 195 | 177 |
| 1902 | Split | J. Kolombatovic | Contribuzione alla fauna dei vertebrati della Dalmazia | 15 | 8 |
| 1912 | Venice | E. Ninni | Catalogo dei pesci del mare Adriatico | 238 | 206 |
| 1913 | Trieste | G. Pastrovic | Manuale del pescatore per l'anno 1913 | 76 | 70 |
| 1917 | Venice | E. Ninni | La pesca nel Mare Adriatico | 85 | 69 |
| 1920 | Venice | E. Ninni | Pesci, crostacei e molluschi nel vernacolo veneziano | 161 | 132 |
| 1928 | Venice | A. Vatova | Compendio della flora e fauna del Mare Adriatico presso Rovigno con la distribuzione geografica delle specie bentoniche | 127 | 122 |
| 1931 | Rome | Ministero dell'Agricoltura e delle Foreste | La pesca nei mari e nelle acque interne d'Italia: notiziario tecnico e legislativo e repertorio della industria e del commercio dei prodotti pescherecci | 28 | 27 |
| 1936 | Rijeka | R. Cella | Il pescatore dilettante. Lo sport della pesca nell'Alto Adriatico | 64 | 64 |
| 1938 | Venice | E. Ninni | Giunte e correzioni ai nomi dialettali dei pesci dati dal Dott. G.D. Nardo nel suo "Prodromus Observationum et Disquisitionum Adriaticae Ichtyologiae" | 57 | 46 |
| 1956 | Bologna | E. Tortonese | Fauna d'Italia | 322 | 237 |
