# Supplementary material for: Coding Early Naturalists' Accounts into Long-Term Fish Community Changes in the Adriatic Sea (1800–2000)
Source: PLoS One. 2010 Nov 17;5(11):e15502. doi: 10.1371/journal.pone.0015502 (PMC2984504; doi:10.1371/journal.pone.0015502)
Supplement: Table S2 — Sources of landing data. (DOC) [file pone.0015502.s004.doc]

Table S2. Sources of landing data.

| Years | Source | Market/area | N° species (min-max) |
| --- | --- | --- | --- |
| 1874-1877 | Statistische Monatsschrift | Austro-Hungarian littoral | 15-88 |
| 1879 | Faber - The fisheries of the Adriatic and the fish thereof | Austro-Hungarian littoral | 50 |
| 1880 | De Marchesetti – La pesca lungo le coste orientali dell’Adria | Austro-Hungarian littoral | 46 |
| 1902-14, 1919-35, 1938-39, 1950-68 | Bollettino Statistico del Comune di Trieste; Dati Statistici del Comune di Trieste; Rivista Mensile della Città di Trieste | Trieste | 60-91 |
| 1904-05 | Austria - Archiv fur volkswirtschaftliche Gesetzgebung und Statistik, fur Industrie, Handel, Verlehr und Patentwesen | Austro-Hungarian littoral | 27 |
| 1904-1917, 1919-32, | D’Ancona – Dell’influenza della stasi peschereccia del periodo 1914-18 sul patrimonio ittico dell’Alto Adriatico | Trieste | 51-76 |
| 1905, 1919-24 | D’Ancona – Dell’influenza della stasi peschereccia del periodo 1914-18 sul patrimonio ittico dell’Alto Adriatico | Venice | 33-53 |
| 1905-09 | 22° Congresso Generale della Società Austriaca di Pesca e Piscicultura Marina | Trieste | 53 |
| 1905, 1925-27 | I prodotti delle aque sul mercato di Venezia | Venice | 36-55 |
| 1909-1910 | Annuario Marittimo per l’anno 1912 compilato a cura dell’I.R. Governo Marittimo | Austro-Hungarian littoral | 27 |
| 1914-32 | D’Ancona – Dell’influenza della stasi peschereccia del periodo 1914-18 sul patrimonio ittico dell’Alto Adriatico | Rijeka | 39-62 |
| 1948 | Consorzio Territoriale per la Tutela della Pesca | Istria | 52 |
| 1948, 1954, 1957-69 | Consorzio Territoriale per la Tutela della Pesca | Gulf of Trieste | 38-54 |
| 1953-54 | Statistica della Pesca e della Caccia | Porto Corsini | 28-33 |
| 1953-72 | Statistica della Pesca e della Caccia; Annuario Statistico della Pesca e della Caccia | Chioggia | 20-36 |
| 1953-72 | Statistica della Pesca e della Caccia; Annuario Statistico della Pesca e della Caccia | Trieste | 19-44 |
| 1953-72 | Statistica della Pesca e della Caccia; Annuario Statistico della Pesca e della Caccia | Venice | 19-56 |
| 1955-72 | Statistica della Pesca e della Caccia; Annuario Statistico della Pesca e della Caccia | Ravenna | 18-32 |
| 1956-57 | Statistica della Pesca e della Caccia | Cattolica | 30-32 |
| 1956-57 | Statistica della Pesca e della Caccia | Cesenatico | 24-23 |
| 1956-57 | Statistica della Pesca e della Caccia | Grado | 35-39 |
| 1956-57 | Statistica della Pesca e della Caccia | Porto Garibaldi | 18-27 |
| 1957 | Statistica della Pesca e della Caccia | Caorle | 18 |
| 1957 | Statistica della Pesca e della Caccia | Goro | 22 |
| 1957 | Statistica della Pesca e della Caccia | Rimini | 35 |
| 1958-72 | Statistica della Pesca e della Caccia; Annuario Statistico della Pesca e della Caccia | Monfalcone | 19-28 |
| 1974-2000 | Annuario Statistico della Zootecnia, Pesca e Caccia; Statistiche della Zootecnia, Pesca e Caccia; Statistiche della Caccia e della Pesca; Statistiche della Pesca e della Caccia; Statistiche sulla Pesca, Caccia e Zootecnia | Emilia Romagna | 21-32 |
| 1974-2000 | Annuario Statistico della Zootecnia, Pesca e Caccia; Statistiche della Zootecnia, Pesca e Caccia; Statistiche della Caccia e della Pesca; Statistiche della Pesca e della Caccia; Statistiche sulla Pesca, Caccia e Zootecnia | Friuli Venezia Giulia | 18-32 |
| 1974-2000 | Annuario Statistico della Zootecnia, Pesca e Caccia; Statistiche della Zootecnia, Pesca e Caccia; Statistiche della Caccia e della Pesca; Statistiche della Pesca e della Caccia; Statistiche sulla Pesca, Caccia e Zootecnia | Veneto | 24-32 |
| 1945-2000 | Fish market statistics | Chioggia | 8-33 |
| 1989-2000 | Fish market statistics | Trieste | 24-28 |
| 1946-2000 | Fish market statistics | Venice | 8-35 |
