# Supplementary material for: Coding Early Naturalists' Accounts into Long-Term Fish Community Changes in the Adriatic Sea (1800–2000)
Source: PLoS One. 2010 Nov 17;5(11):e15502. doi: 10.1371/journal.pone.0015502 (PMC2984504; doi:10.1371/journal.pone.0015502)
Supplement: Table S3 — Taxonomic groups for which landing statistics were available for the periods 1876–1900, 1901–1925 and 1926–1950. (DOC) [file pone.0015502.s005.doc]

Table S3. Taxonomic groups for which landing statistics were available for the periods 1876–1900, 1901–1925 and 1926–1950.

| Taxonomic group | Species |
| --- | --- |
| Acipenseridae | *Acipenser naccarii, Acipenser sturio, Huso huso* |
| Blenniidae | *Aidablennius sphynx, Blennius ocellaris, Coryphoblennius galerita, Parablennius gattorugine, Parablennius rouxi, Parablennius sanguinolentus, Parablennius tentacularis, Salaria pavo* |
| *Alopias vulpinus* | *Alopias vulpinus* |
| *Alosa* spp. | *Alosa alosa, Alosa fallax* |
| *Anguilla anguilla* | *Anguilla anguilla* |
| *Aphanius fasciatus* | *Aphanius fasciatus* |
| *Aphia minuta* | *Aphia minuta* |
| *Argentina sphyraena* | *Argentina sphyraena* |
| *Arnoglossus laterna* | *Arnoglossus laterna* |
| Triglidae | *Aspitrigla cuculus, Chelidonichthys lastoviza, Eutrigla gurnardus, Lepidotrigla cavillone, Trigla lyra, Chelidonichthys lucerna* |
| *Atherina* spp. | *Atherina boyeri, Atherina hepsetus* |
| *Auxis rochei rochei* | *Auxis rochei rochei* |
| *Belone belone* | *Belone belone* |
| *Boops boops* | *Boops boops* |
| *Carcharhinus plumbeus* | *Carcharhinus plumbeus* |
| *Carcharodon carcharias* | *Carcharodon carcharias* |
| *Centrolophus niger* | *Centrolophus niger* |
| *Cepola macrophthalma* | *Cepola macrophthalma* |
| Mugilidae | *Chelon labrosus, Liza aurata, Liza ramado, Liza saliens, Mugil cephalus* |
| *Chromis chromis* | *Chromis chromis* |
| *Citharus linguatula* | *Citharus linguatula* |
| *Conger conger* | *Conger conger* |
| *Coris julis* | *Coris julis* |
| *Coryphaena hippurus* | *Coryphaena hippurus* |
| *Dasyatis* spp. | *Dasyatis centroura, Dasyatis pastinaca* |
| *Dentex* spp. | *Dentex dentex, Dentex gibbosus* |
| *Dicentrarchus labrax* | *Dicentrarchus labrax* |
| *Diplodus* spp. | *Diplodus annularis, Diplodus puntazzo, Diplodus sargus sargus, Diplodus vulgaris* |
| *Dipturus* spp. | *Dipturus batis, Dipturus oxyrinchus* |
| *Echelus myrus* | *Echelus myrus* |
| Trachinidae | *Echiichthys vipera, Trachinus draco, Trachinus radiatus* |
| *Engraulis encrasicolus* | *Engraulis encrasicolus* |
| *Epinephelus marginatus/Polyprion americanus* | *Epinephelus marginatus, Polyprion americanus* |
| *Thunnus* spp. | *Euthynnus alletteratus, Thunnus alalunga, Thunnus thynnus* |
| *Exocoetus volitans* | *Exocoetus volitans* |
| *Gaidropsarus mediterraneus* | *Gaidropsarus mediterraneus* |
| *Gaidropsarus vulgaris* | *Gaidropsarus vulgaris* |
| *Galeorhinus galeus* | *Galeorhinus galeus* |
| *Gasterosteus aculeatus aculeatus* | *Gasterosteus aculeatus aculeatus* |
| *Gobius* spp. | *Gobius auratus, Gobius cobitis, Gobius cruentatus, Gobius geniporus, Gobius niger, Gobius paganellus* |
| *Isurus oxyrinchus* | *Isurus oxyrinchus* |
| *Katsuwonus pelamis* | *Katsuwonus pelamis* |
| Rajidae | *Leucoraja fullonica, Raja asterias, Raja clavata, Raja miraletus, Raja radula, Rostroraja alba* |
| *Lichia amia/Tracinotus ovatus* | *Lichia amia, Traconotus ovatus* |
| *Lithognathus mormyrus* | *Lithognathus mormyrus* |
| *Lophius* spp. | *Lophius budegassa, Lophius piscatorius* |
| *Luvarus imperialis* | *Luvarus imperialis* |
| *Merlangius merlangus* | *Merlangius merlangus* |
| *Merluccius merluccius* | *Merluccius merluccius* |
| *Micromesistius poutassou* | *Micromesistius poutassou* |
| *Mullus* spp. | *Mullus barbatus barbatus, Mullus surmuletus* |
| *Muraena helena* | *Muraena helena* |
| *Mustelus* spp. | *Mustelus asterias, Mustelus mustelus* |
| *Myliobatis aquila/Pteromylaeus bovinus* | *Myliobatis aquila, Pteromylaeus bovinus* |
| *Naucrates ductor* | *Naucrates ductor* |
| *Oblada melanura* | *Oblada melanura* |
| *Ophidion barbatum* | *Ophidion barbatum* |
| *Oxynotus centrina* | *Oxynotus centrina* |
| *Pagellus* spp. | *Pagellus acarne, Pagellus bogaraveo, Pagellus erythrinus* |
| *Pagrus pagrus* | *Pagrus pagrus* |
| Soleidae | *Pegusa lascaris, Solea solea* |
| *Petromyzon marinus* | *Petromyzon marinus* |
| *Phycis blennoides* | *Phycis blennoides* |
| *Platichthys flesus* | *Platichthys flesus* |
| *Prionace glauca* | *Prionace glauca* |
| *Psetta maxima/Scophtalmus rhombus* | *Psetta maxima, Scophtalmus rhombus* |
| *Sarda sarda* | *Sarda sarda* |
| *Sardina pilchardus* | *Sardina pilchardus* |
| *Sardinella aurita* | *Sardinella aurita* |
| *Sarpa salpa* | *Sarpa salpa* |
| *Sciaena umbra* | *Sciaena umbra* |
| *Scomber* spp. | *Scomber japonicus, Scomber scombrus* |
| *Scorpaena* spp. | *Scorpaena porcus, Scorpaena scrofa* |
| *Scyliorhinus* spp. | *Scyliorhinus canicula, Scyliorhinus stellaris* |
| *Seriola dumerili* | *Seriola dumerili* |
| *Serranus cabrilla* | *Serranus cabrilla* |
| *Serranus hepatus* | *Serranus hepatus* |
| *Serranus scriba* | *Serranus scriba* |
| *Sparus aurata* | *Sparus aurata* |
| *Sphyraena sphyraena* | *Sphyraena sphyraena* |
| *Sphyrna* spp. | *Sphyrna tudes, Sphyrna zygaena* |
| *Spicara* spp. | *Spicara maena, Spicara smaris* |
| *Spondyliosoma cantharus* | *Spondyliosoma cantharus* |
| *Sprattus sprattus sprattus* | *Sprattus sprattus sprattus* |
| *Squalus* spp. | *Squalus acanthias, Squalus blainville,* |
| *Squatina* spp. | *Squatina squatina, Squatina oculata* |
| *Stromateus fiatola* | *Stromateus fiatola* |
| *Symphodus* spp. | *Symphodus cinereus, Symphodus mediterraneus, Symphodus melanocercus, Symphodus melops, Symphodus ocellatus, Symphodus roissali, Symphodus rostratus, Symphodus tinca* |
| *Syngnathus typhle* | *Syngnathus typhle* |
| *Torpedo marmorata* | *Torpedo marmorata* |
| *Trachurus* spp. | *Trachurus trachurus, Trachurus mediterraneus* |
| *Trisopterus minutus* | *Trisopterus minutus* |
| *Umbrina cirrosa* | *Umbrina cirrosa* |
| *Uranoscopus scaber* | *Uranoscopus scaber* |
| *Xiphias gladius* | *Xiphias gladius* |
| *Zeus faber* | *Zeus faber* |
| *Zosterisessor ophiocephalus* | *Zosterisessor ophiocephalus* |
