# Supplementary material for: Coding Early Naturalists' Accounts into Long-Term Fish Community Changes in the Adriatic Sea (1800–2000)
Source: PLoS One. 2010 Nov 17;5(11):e15502. doi: 10.1371/journal.pone.0015502 (PMC2984504; doi:10.1371/journal.pone.0015502)
Supplement: Table S4 — Class limits (median and interquartile range) for each period of intercalibration and for the entire period with overlapping information that discriminate the qualitative classes of perceived abundance. (DOC) [file pone.0015502.s006.doc]

Table S4. Class limits (median and interquartile range) for each period of intercalibration and for the entire period with overlapping information that discriminate the qualitative classes of perceived abundance.

|  | Very rare/rare (%) | Rare/common (%) | Common/very common (%) |
| --- | --- | --- | --- |
| 1876-1900 | 0.00624 (0.00619-0.00628) | 0.152 (0.150-0.155) | 12.67 (12.64-12.70) |
| 1901-1925 | 0.0016 (0.0011-0.0017) | 0.037 (0.033-0.04) | 1.28 (1.27-1.32) |
| 1926-1950 | 0.0012 (0.0011-0.0012) | 0.016 (0.016-0.017) | 18.31 (18.27-18.32) |
| 1876-1950 (median)[[1]](#footnote-2) | 0.002 (0.001-0.006) | 0.037 (0.004-5.929) | 12.6 (1.3-18.3) |

1. Global class limits: median value of the whole set of limits, *N* = 3000. [↑](#footnote-ref-2)
