# Supplementary material for: Coding Early Naturalists' Accounts into Long-Term Fish Community Changes in the Adriatic Sea (1800–2000)
Source: PLoS One. 2010 Nov 17;5(11):e15502. doi: 10.1371/journal.pone.0015502 (PMC2984504; doi:10.1371/journal.pone.0015502)
Supplement: Table S5 — Class weights, computed using the intercalibration, and their association with classes of perceived abundance. (DOC) [file pone.0015502.s007.doc]

Table S5. Class weights, computed using the intercalibration, and their association with classes of perceived abundance.

| Perceived abundance | Class weights (median and interquartile range) |
| --- | --- |
| very rare | 0.0004 (0.0003-0.0007) |
| rare | 0.007 (0.004-0.030) |
| common | 0.5 (0.2-1.4) |
| very common | 13.7 (5.9-18.3) |
