# Supplementary material for: Coding Early Naturalists' Accounts into Long-Term Fish Community Changes in the Adriatic Sea (1800–2000)
Source: PLoS One. 2010 Nov 17;5(11):e15502. doi: 10.1371/journal.pone.0015502 (PMC2984504; doi:10.1371/journal.pone.0015502)
Supplement: Table S6 — List of species described in naturalists' documents and the species' ecological characteristics according to Fishbase. (DOC) [file pone.0015502.s008.doc]

Table S6. List of species described in naturalists’ documents and the species’ ecological characteristics according to Fishbase.

| Family | Scientific name | Phylum | Functional group | L_max_ (cm)**[[1]](#footnote-2)** | TL**[[2]](#footnote-3)** | Age at sexual maturity (years) |
| --- | --- | --- | --- | --- | --- | --- |
| Labridae | *Acantholabrus palloni* | Actinopterygies |  | 25 | 3.5 | 2.5 |
| Acipenseridae | *Acipenser naccarii* | Actinopterygies | large demersals | 200 | 3.4 | 9.3 |
| Acipenseridae | *Acipenser sturio* | Actinopterygies | large demersals | 500 | 3.5 | 16.7 |
| Blenniidae | *Aidablennius sphynx* | Actinopterygies |  | 8 | 2.5 | 1 |
| Alopiidae | *Alopias vulpinus* | Chondrichthyes | large pelagics | 760 | 4.5 | 4.9 |
| Clupeidae | *Alosa alosa* | Actinopterygies | medium pelagics | 83 | 3.6 | 1.7 |
| Clupeidae | *Alosa fallax* | Actinopterygies | medium pelagics | 60 | 3.6 | 1.7 |
| Ammodytidae | *Ammodytes tobianus* | Actinopterygies | small demersals | 20 | 3.2 | 1.3 |
| Anguillidae | *Anguilla anguilla* | Actinopterygies | large demersals | 133 | 3.5 | 11.9 |
| Serranidae | *Anthias anthias* | Actinopterygies |  | 27 | 3.8 | 2.4 |
| Cyprinodontidae | *Aphanius fasciatus* | Actinopterygies | small demersals | 6 | 2.7 | 2.2 |
| Gobiidae | *Aphia minuta* | Actinopterygies | small pelagics | 7.9 | 3.1 | 0.6 |
| Apogonidae | *Apogon imberbis* | Actinopterygies |  | 15 | 3.9 | 0.9 |
| Argentinidae | *Argentina sphyraena* | Actinopterygies | medium demersals | 35 | 3.6 | 1.7 |
| Sciaenidae | *Argyrosomus regius* | Actinopterygies | large demersals | 230 | 4.3 | 6.2 |
| Bothidae | *Arnoglossus kessleri* | Actinopterygies | small demersals | 10 | 4 | 0.8 |
| Bothidae | *Arnoglossus laterna* | Actinopterygies | small demersals | 25 | 3.6 | 0.8 |
| Triglidae | *Aspitrigla cuculus* | Actinopterygies | medium demersals | 50 | 3.8 | 1 |
| Atherinidae | *Atherina boyeri* | Actinopterygies | small demersals | 20 | 2.3 | 1 |
| Atherinidae | *Atherina hepsetus* | Actinopterygies | small pelagics | 20 | 3.2 | 1.8 |
| Scombridae | *Auxis rochei rochei* | Actinopterygies | medium pelagics | 50 | 4.1 | 1.7 |
| Balistidae | *Balistes capriscus* | Actinopterygies |  | 60 | 3.4 | 1.8 |
| Belonidae | *Belone belone* | Actinopterygies | large pelagics | 93 | 4.2 | 2.6 |
| Blenniidae | *Blennius ocellaris* | Actinopterygies |  | 20 | 3.5 | 2 |
| Sparidae | *Boops boops* | Actinopterygies |  | 36 | 3 | 2.6 |
| Bothidae | *Bothus podas* | Actinopterygies | medium demersals | 45 | 3.4 | 1.1 |
| Bramidae | *Brama brama* | Actinopterygies | large pelagics | 100 | 4.1 | 8.1 |
| Soleidae | *Buglossidium luteum* | Actinopterygies | small demersals | 15 | 3.3 | 1.6 |
| Callionymidae | *Callionymus lyra* | Actinopterygies | small demersals | 30 | 3.3 | 1.7 |
| Callionymidae | *Callionymus maculatus* | Actinopterygies | small demersals | 16 | 3.2 | 1.3 |
| Callionymidae | *Callionymus pusillus* | Actinopterygies | small demersals | 14 | 3.3 | 1.1 |
| Callionymidae | *Callionymus risso* | Actinopterygies | small demersals | 11 | 3 | 1 |
| Carangidae | *Campogramma glaycos* | Actinopterygies | medium pelagics | 60 | 4.5 | 2.3 |
| Caproidae | *Capros aper* | Actinopterygies | small demersals | 30 | 3.1 |  |
| Carapidae | *Carapus acus* | Actinopterygies | small demersals | 20.8 | 4 |  |
| Carcharhinidae | *Carcharhinus plumbeus* | Chondrichthyes | large pelagics | 250 | 4.5 | 9 |
| Odontaspididae | *Carcharias taurus* | Chondrichthyes | large demersals | 320 | 4.5 | 3.8 |
| Lamnidae | *Carcharodon carcharias* | Chondrichthyes | large pelagics | 720 | 4.5 | 8 |
| Centrolophidae | *Centrolophus niger* | Actinopterygies | large pelagics | 150 | 4 | 8.3 |
| Cepolidae | *Cepola macrophthalma* | Actinopterygies | medium demersals | 80 | 3.1 | 3.7 |
| Cetorhinidae | *Cetorhinus maximus* | Chondrichthyes | large pelagics | 900 | 3.2 | 7.8 |
| Triglidae | *Chelidonichthys lastoviza* | Actinopterygies | medium demersals | 40 | 3.4 | 1.9 |
| Triglidae | *Chelidonichthys lucernus* | Actinopterygies | medium demersals | 75 | 3.6 | 4.3 |
| Mugilidae | *Chelon labrosus* | Actinopterygies | medium pelagics | 75 | 2.4 | 5.4 |
| Pomacentridae | *Chromis chromis* | Actinopterygies |  | 25 | 3 | 3.2 |
| Gobiidae | *Chromogobius quadrivittatus* | Actinopterygies |  | 6.6 | 3.4 | 1.3 |
| Citharidae | *Citharus linguatula* | Actinopterygies | small demersals | 30 | 4 | 3 |
| Clinidae | *Clinitrachus argentatus* | Actinopterygies |  | 10 | 3.5 |  |
| Congridae | *Conger conger* | Actinopterygies |  | 300 | 4.3 | 9.1 |
| Labridae | *Coris julis* | Actinopterygies |  | 30 | 3.2 | 6.6 |
| Coryphaenidae | *Coryphaena hippurus* | Actinopterygies | large pelagics | 210 | 4.4 | 0.4 |
| Blenniidae | *Coryphoblennius galerita* | Actinopterygies |  | 7.6 | 2.2 | 1.2 |
| Dactylopteridae | *Dactylopterus volitans* | Actinopterygies | medium demersals | 90 | 3.6 |  |
| Ophichthidae | *Dalophis imberbis* | Actinopterygies | large demersals | 150 | 4 | 3.7 |
| Dasyatidae | *Dasyatis centroura* | Chondrichthyes | large demersals | 220 | 3.8 | 5.6 |
| Dasyatidae | *Dasyatis pastinaca* | Chondrichthyes | medium demersals | 57 | 4 | 6.6 |
| Gobiidae | *Deltentosteus quadrimaculatus* | Actinopterygies | small demersals | 8 | 3.1 | 1.3 |
| Sparidae | *Dentex dentex* | Actinopterygies | large demersals | 100 | 4.5 | 6.3 |
| Sparidae | *Dentex gibbosus* | Actinopterygies | large demersals | 106 | 4.1 | 4.1 |
| Moronidae | *Dicentrarchus labrax* | Actinopterygies | large demersals | 103 | 3.8 | 3 |
| Gobiesocidae | *Diplecogaster bimaculata bimaculata* | Actinopterygies | small demersals | 6 | 3.3 | 2.8 |
| Sparidae | *Diplodus annularis* | Actinopterygies |  | 24 | 3.4 | 3 |
| Sparidae | *Diplodus puntazzo* | Actinopterygies |  | 60 | 2.9 | 1.6 |
| Sparidae | *Diplodus sargus sargus* | Actinopterygies |  | 45 | 3 | 6.6 |
| Sparidae | *Diplodus vulgaris* | Actinopterygies |  | 45 | 3.2 | 1.9 |
| Rajidae | *Dipturus batis* | Chondrichthyes | large demersals | 285 | 4 | 9 |
| Rajidae | *Dipturus oxyrinchus* | Chondrichthyes | large demersals | 150 | 3.5 | 5.8 |
| Ophichthidae | *Echelus myrus* | Actinopterygies | large demersals | 100 |  | 2.7 |
| Trachinidae | *Echiichthys vipera* | Actinopterygies | small demersals | 15 | 4.4 |  |
| Echinorhinidae | *Echinorhinus brucus* | Chondrichthyes | large demersals | 310 | 4.4 |  |
| Engraulidae | *Engraulis encrasicolus* | Actinopterygies | small pelagics | 20 | 3.1 | 1.4 |
| Serranidae | *Epinephelus marginatus* | Actinopterygies |  | 150 | 3.7 | 6.7 |
| Dalatiidae | *Etmopterus spinax* | Chondrichthyes | medium demersals | 60 | 3.8 |  |
| Scombridae | *Euthynnus alletteratus* | Actinopterygies | large pelagics | 122 | 4.5 | 3.2 |
| Triglidae | *Eutrigla gurnardus* | Actinopterygies | medium demersals | 60 | 3.6 | 4.3 |
| Exocoetidae | *Exocoetus volitans* | Actinopterygies | small pelagics | 30 | 3 | 0.6 |
| Lotidae | *Gaidropsarus mediterraneus* | Actinopterygies |  | 50 | 3.4 | 3.9 |
| Lotidae | *Gaidropsarus vulgaris* | Actinopterygies | medium demersals | 60 | 3.2 | 1.4 |
| Triakidae | *Galeorhinus galeus* | Chondrichthyes | large demersals | 193 | 4.2 | 3.4 |
| Scyliorhinidae | *Galeus melastomus* | Chondrichthyes | medium demersals | 75 | 4.2 | 3.3 |
| Gasterosteidae | *Gasterosteus aculeatus aculeatus* | Actinopterygies | small pelagics | 11 | 3.5 | 0.5 |
| Gobiidae | *Gobius auratus* | Actinopterygies |  | 10 | 3 | 1.5 |
| Gobiidae | *Gobius cobitis* | Actinopterygies |  | 27 | 3 | 3.4 |
| Gobiidae | *Gobius cruentatus* | Actinopterygies |  | 18 | 3.1 | 2.3 |
| Gobiidae | *Gobius geniporus* | Actinopterygies | small demersals | 16 | 3.3 | 2.1 |
| Gobiidae | *Gobius niger* | Actinopterygies | small demersals | 18 | 3.2 | 2.7 |
| Gobiidae | *Gobius paganellus* | Actinopterygies | small demersals | 12 | 3.3 | 1.1 |
| Gobiidae | *Gobiusculus flavescens* | Actinopterygies |  | 6 | 3.2 | 1 |
| Gobiesocidae | *Gouania willdenowi* | Actinopterygies |  | 5 |  | 2 |
| Bythitidae | *Grammonus ater* | Actinopterygies |  | 12 | 3.5 | 1.3 |
| Ammodytidae | *Gymnammodytes cicerelus* | Actinopterygies | small demersals | 17 | 3.4 | 1.3 |
| Muraenidae | *Gymnothorax unicolor* | Actinopterygies |  | 100 | 3.4 |  |
| Gymnuridae | *Gymnura altavela* | Chondrichthyes | large demersals | 400 | 4.5 |  |
| Sebastidae | *Helicolenus dactylopterus dactylopterus* | Actinopterygies | medium demersals | 47 | 3.8 | 7.1 |
| Hexanchidae | *Heptranchias perlo* | Chondrichthyes | large demersals | 137 | 4.2 |  |
| Hexanchidae | *Hexanchus griseus* | Chondrichthyes | large demersals | 482 | 4.3 |  |
| Syngnathidae | *Hippocampus guttulatus* | Actinopterygies |  | 16 | 3.5 | 1 |
| Syngnathidae | *Hippocampus hippocampus* | Actinopterygies |  | 15 | 3.2 | 0.9 |
| Exocoetidae | *Hirundichthys rondeletii* | Actinopterygies | small pelagics | 30 |  | 0.6 |
| Acipenseridae | *Huso huso* | Actinopterygies | large demersals | 500 | 4.1 | 25 |
| Lamnidae | *Isurus oxyrinchus* | Chondrichthyes | large pelagics | 400 | 4.5 | 2.6 |
| Scombridae | *Katsuwonus pelamis* | Actinopterygies | large pelagics | 108 | 4.4 | 1 |
| Gobiidae | *Knipowitschia panizzae* | Actinopterygies | small demersals | 5.5 | 3.5 | 0.9 |
| Labridae | *Labrus merula* | Actinopterygies |  | 45 | 3.2 | 3.1 |
| Labridae | *Labrus mixtus* | Actinopterygies |  | 40 | 3.9 | 5.5 |
| Labridae | *Labrus viridis* | Actinopterygies |  | 47 | 3.8 | 4.8 |
| Lamnidae | *Lamna nasus* | Chondrichthyes | large pelagics | 350 | 4.5 | 8.8 |
| Petromyzontidae | *Lampetra fluviatilis* | Actinopterygies | medium demersals | 50 | 4.5 | 5.7 |
| Gobiesocidae | *Lepadogaster candolii* | Actinopterygies |  | 7.5 | 2.8 | 3.3 |
| Gobiesocidae | *Lepadogaster lepadogaster* | Actinopterygies |  | 6.5 |  | 2.9 |
| Trichiuridae | *Lepidopus caudatus* | Actinopterygies | large demersals | 210 | 3.8 | 1.9 |
| Triglidae | *Lepidotrigla cavillone* | Actinopterygies | small demersals | 20 | 3.2 | 1.6 |
| Gobiidae | *Lesueurigobius suerii* | Actinopterygies | small demersals | 5 | 3.6 | 0.9 |
| Rajidae | *Leucoraja fullonica* | Chondrichthyes | large demersals | 120 | 3.5 | 5 |
| Carangidae | *Lichia amia* | Actinopterygies | large pelagics | 200 | 4.5 | 5.6 |
| Blenniidae | *Lipophrys pholis* | Actinopterygies |  | 30 | 3.1 | 1.6 |
| Sparidae | *Lithognathus mormyrus* | Actinopterygies | medium demersals | 55 | 3.4 | 2.9 |
| Mugilidae | *Liza aurata* | Actinopterygies | medium pelagics | 59 | 2.5 | 3.3 |
| Mugilidae | *Liza ramado* | Actinopterygies | medium pelagics | 70 | 2.2 | 4 |
| Mugilidae | *Liza saliens* | Actinopterygies | medium pelagics | 40 | 2.2 | 3.7 |
| Lophiidae | *Lophius budegassa* | Actinopterygies | large demersals | 100 | 4.5 | 7.7 |
| Lophiidae | *Lophius piscatorius* | Actinopterygies | large demersals | 200 | 4.4 | 7.1 |
| Lophotidae | *Lophotus lacepede* | Actinopterygies | large pelagics | 200 | 4.5 |  |
| Luvaridae | *Luvarus imperialis* | Actinopterygies | large pelagics | 200 | 4 |  |
| Centriscidae | *Macroramphosus scolopax* | Actinopterygies | small demersals | 20 | 3.5 | 1.7 |
| Gadidae | *Merlangius merlangus* | Actinopterygies | medium demersals | 70 | 4.4 | 3.9 |
| Merlucciidae | *Merluccius merluccius* | Actinopterygies | large demersals | 140 | 4.4 | 6.2 |
| Soleidae | *Microchirus ocellatus* | Actinopterygies | small demersals | 20 |  | 1.6 |
| Soleidae | *Microchirus variegatus* | Actinopterygies | medium demersals | 35 | 3.3 | 3.1 |
| Gadidae | *Micromesistius poutassou* | Actinopterygies | medium pelagics | 50 | 4 | 3.8 |
| Myliobatidae | *Mobula mobular* | Chondrichthyes | large pelagics | 520 | 3.7 | 3 |
| Molidae | *Mola mola* | Actinopterygies | large pelagics | 333 | 3.9 |  |
| Soleidae | *Monochirus hispidus* | Actinopterygies | small demersals | 20 |  | 2 |
| Mugilidae | *Mugil cephalus* | Actinopterygies | large pelagics | 120 | 2.1 | 5.6 |
| Mullidae | *Mullus barbatus barbatus* | Actinopterygies | small demersals | 30 | 3.2 | 3 |
| Mullidae | *Mullus surmuletus* | Actinopterygies | medium demersals | 40 | 3.4 | 2.8 |
| Muraenidae | *Muraena helena* | Actinopterygies |  | 150 | 4.2 |  |
| Triakidae | *Mustelus asterias* | Chondrichthyes | large demersals | 140 | 3.7 | 2 |
| Triakidae | *Mustelus mustelus* | Chondrichthyes | large demersals | 200 | 3.8 | 4.9 |
| Myliobatidae | *Myliobatis aquila* | Chondrichthyes | large demersals | 183 | 3.6 | 3.6 |
| Carangidae | *Naucrates ductor* | Actinopterygies | small pelagics | 70 | 4 | 0.3 |
| Syngnathidae | *Nerophis maculatus* | Actinopterygies | small demersals | 30 | 3.4 | 1.5 |
| Syngnathidae | *Nerophis ophidion* | Actinopterygies |  | 29 | 4 | 0.7 |
| Sparidae | *Oblada melanura* | Actinopterygies |  | 34 | 3 | 2.4 |
| Ophidiidae | *Ophidion barbatum* | Actinopterygies | small demersals | 25 | 3.6 | 1.7 |
| Ophichthidae | *Ophisurus serpens* | Actinopterygies | large demersals | 250 |  | 5.5 |
| Scombridae | *Orcynopsis unicolor* | Actinopterygies | large pelagics | 130 | 4.5 | 2.8 |
| Dalatiidae | *Oxynotus centrina* | Chondrichthyes | large demersals | 150 | 3.1 |  |
| Sparidae | *Pagellus acarne* | Actinopterygies | medium demersals | 36 | 3.5 | 3.4 |
| Sparidae | *Pagellus bogaraveo* | Actinopterygies | medium demersals | 70 | 3.7 | 7.3 |
| Sparidae | *Pagellus erythrinus* | Actinopterygies | medium demersals | 60 | 3.4 | 4.4 |
| Sparidae | *Pagrus pagrus* | Actinopterygies | large demersals | 91 | 3.6 | 4.7 |
| Blenniidae | *Parablennius gattorugine* | Actinopterygies |  | 30 | 2.9 | 1 |
| Blenniidae | *Parablennius rouxi* | Actinopterygies |  | 8 | 2.6 | 1 |
| Blenniidae | *Parablennius sanguinolentus* | Actinopterygies |  | 20 | 2.1 | 2 |
| Blenniidae | *Parablennius tentacularis* | Actinopterygies |  | 15 | 3.1 | 1.6 |
| Blenniidae | *Paralipophrys trigloides* | Actinopterygies |  | 13 | 3.5 | 1.4 |
| Ophidiidae | *Parophidion vassali* | Actinopterygies | small demersals | 25 | 3.3 | 1.7 |
| Soleidae | *Pegusa impar* | Actinopterygies | medium demersals | 35 | 3.2 | 2.6 |
| Soleidae | *Pegusa lascaris* | Actinopterygies | medium demersals | 40 | 3.2 | 1.7 |
| Peristediidae | *Peristedion cataphractum* | Actinopterygies | medium demersals | 40 |  | 3.9 |
| Petromyzontidae | *Petromyzon marinus* | Actinopterygies | large demersals | 120 | 4.4 | 6.8 |
| Phycidae | *Phycis blennoides* | Actinopterygies | large demersals | 110 | 3.7 | 3.9 |
| Phycidae | *Phycis phycis* | Actinopterygies | medium demersals | 65 | 4.3 | 3.4 |
| Pleuronectidae | *Platichthys flesus* | Actinopterygies | medium demersals | 60 | 3.2 | 3 |
| Polyprionidae | *Polyprion americanus* | Actinopterygies |  | 210 | 3.8 | 12 |
| Pomatomidae | *Pomatomus saltatrix* | Actinopterygies | large pelagics | 130 | 4.5 | 3.3 |
| Gobiidae | *Pomatoschistus minutus* | Actinopterygies | small demersals | 11 | 3.2 | 0.9 |
| Gobiidae | *Pomatoschistus quagga* | Actinopterygies | small demersals | 6 | 3.3 | 1 |
| Carcharhinidae | *Prionace glauca* | Chondrichthyes | large pelagics | 400 | 4.2 | 3 |
| Scophthalmidae | *Psetta maxima* | Actinopterygies | large demersals | 100 | 4 | 1.2 |
| Carangidae | *Pseudocaranx dentex* | Actinopterygies | large demersals | 122 | 3.9 | 10 |
| Myliobatidae | *Pteromylaeus bovinus* | Chondrichthyes | large demersals | 250 | 3.8 | 5.5 |
| Rajidae | *Raja asterias* | Chondrichthyes | medium demersals | 70 | 3.5 | 3.1 |
| Rajidae | *Raja clavata* | Chondrichthyes | large demersals | 105 | 3.8 | 3 |
| Rajidae | *Raja miraletus* | Chondrichthyes | medium demersals | 63 | 3.8 | 2.8 |
| Rajidae | *Raja radula* | Chondrichthyes | medium demersals | 70 | 3.7 | 3.1 |
| Molidae | *Ranzania laevis* | Actinopterygies | large pelagics | 100 | 3.7 |  |
| Echeneidae | *Remora remora* | Actinopterygies | medium pelagics | 86.4 | 3.1 |  |
| Myliobatidae | *Rhinoptera marginata* | Chondrichthyes | large demersals | 200 | 3.8 | 2.4 |
| Rajidae | *Rostroraja alba* | Chondrichthyes | large demersals | 230 | 4.4 | 7.9 |
| Blenniidae | *Salaria pavo* | Actinopterygies |  | 13 | 2.9 | 1.6 |
| Scombridae | *Sarda sarda* | Actinopterygies | large pelagics | 91.4 | 4.5 | 1 |
| Clupeidae | *Sardina pilchardus* | Actinopterygies | small pelagics | 25 | 2.6 | 1.7 |
| Clupeidae | *Sardinella aurita* | Actinopterygies | medium pelagics | 31 | 3 | 2.7 |
| Sparidae | *Sarpa salpa* | Actinopterygies |  | 51 | 2 | 3.4 |
| Centrolophidae | *Schedophilus ovalis* | Actinopterygies | large demersals | 100 | 3.5 | 5.1 |
| Sciaenidae | *Sciaena umbra* | Actinopterygies |  | 70 | 3.7 | 5.9 |
| Scombridae | *Scomber japonicus* | Actinopterygies | medium pelagics | 64 | 3.1 | 2.1 |
| Scombridae | *Scomber scombrus* | Actinopterygies | medium pelagics | 60 | 3.6 | 2.5 |
| Scomberesocidae | *Scomberesox saurus saurus* | Actinopterygies | medium pelagics | 50 | 3.6 | 1 |
| Scophthalmidae | *Scophthalmus rhombus* | Actinopterygies | medium demersals | 75 | 3.8 | 5 |
| Scorpaenidae | *Scorpaena porcus* | Actinopterygies |  | 37 | 3.9 | 4.3 |
| Scorpaenidae | *Scorpaena scrofa* | Actinopterygies |  | 50 | 4.3 | 8.2 |
| Scyliorhinidae | *Scyliorhinus canicula* | Chondrichthyes | large demersals | 100 | 3.7 | 4.8 |
| Scyliorhinidae | *Scyliorhinus stellaris* | Chondrichthyes | large demersals | 170 | 4 | 5.2 |
| Carangidae | *Seriola dumerili* | Actinopterygies |  | 190 | 4.5 | 2.4 |
| Serranidae | *Serranus cabrilla* | Actinopterygies | medium demersals | 40 | 3.4 | 2 |
| Serranidae | *Serranus hepatus* | Actinopterygies | small demersals | 25 | 3.5 | 2.2 |
| Serranidae | *Serranus scriba* | Actinopterygies | medium demersals | 36 | 3.8 | 3.1 |
| Soleidae | *Solea solea* | Actinopterygies | medium demersals | 70 | 3.1 | 0.9 |
| Sparidae | *Sparus aurata* | Actinopterygies | medium demersals | 70 | 3.4 | 2.5 |
| Sphyraenidae | *Sphyraena sphyraena* | Actinopterygies | large pelagics | 165 | 4 | 4.8 |
| Sphyrnidae | *Sphyrna tudes* | Chondrichthyes | large pelagics | 134 | 3.6 | 2.6 |
| Sphyrnidae | *Sphyrna zygaena* | Chondrichthyes | large pelagics | 500 | 4.5 | 6.3 |
| Centracanthidae | *Spicara maena* | Actinopterygies | small pelagics | 25 | 4.2 | 2.5 |
| Centracanthidae | *Spicara smaris* | Actinopterygies | small pelagics | 20 | 3 | 2.7 |
| Sparidae | *Spondyliosoma cantharus* | Actinopterygies | medium demersals | 60 | 3.3 | 3.8 |
| Clupeidae | *Sprattus sprattus sprattus* | Actinopterygies | small pelagics | 16 | 3 | 1.6 |
| Squalidae | *Squalus acanthias* | Chondrichthyes | large demersals | 160 | 4.3 | 14.1 |
| Squalidae | *Squalus blainville* | Chondrichthyes | large demersals | 100 | 4 | 6 |
| Squatinidae | *Squatina oculata* | Chondrichthyes | large demersals | 160 | 4 | 4.8 |
| Squatinidae | *Squatina squatina* | Chondrichthyes | large demersals | 183 | 4 | 7.1 |
| Stromateidae | *Stromateus fiatola* | Actinopterygies | medium pelagics | 50 | 3.9 | 2.3 |
| Labridae | *Symphodus cinereus* | Actinopterygies |  | 16 | 3.3 | 2.8 |
| Labridae | *Symphodus mediterraneus* | Actinopterygies |  | 18 | 3.1 | 1.3 |
| Labridae | *Symphodus melanocercus* | Actinopterygies |  | 14 | 3 | 1.6 |
| Labridae | *Symphodus melops* | Actinopterygies |  | 28 | 3.2 | 2.5 |
| Labridae | *Symphodus ocellatus* | Actinopterygies |  | 12 | 3.3 | 0.9 |
| Labridae | *Symphodus roissali* | Actinopterygies |  | 17 | 3.5 | 2.3 |
| Labridae | *Symphodus rostratus* | Actinopterygies |  | 13 | 3.4 | 1.2 |
| Labridae | *Symphodus tinca* | Actinopterygies |  | 44 | 3.1 | 2.9 |
| Cynoglossidae | *Symphurus nigrescens* | Actinopterygies | small demersals | 12 | 3.3 | 1.6 |
| Soleidae | *Synapturichthys kleinii* | Actinopterygies | medium demersals | 40 |  | 2 |
| Syngnathidae | *Syngnathus abaster* | Actinopterygies |  | 21 | 3.2 | 1.1 |
| Syngnathidae | *Syngnathus acus* | Actinopterygies |  | 50 | 3.4 | 2.2 |
| Syngnathidae | *Syngnathus typhle* | Actinopterygies |  | 35 | 4.3 | 1.3 |
| Synodontidae | *Synodus saurus* | Actinopterygies | medium demersals | 40 | 4.5 | 1.5 |
| Istiophoridae | *Tetrapturus belone* | Actinopterygies | large pelagics | 240 | 4.5 | 1.3 |
| Labridae | *Thalassoma pavo* | Actinopterygies |  | 25 | 3.5 | 3.1 |
| Scombridae | *Thunnus alalunga* | Actinopterygies | large pelagics | 140 | 4.3 | 3.9 |
| Scombridae | *Thunnus thynnus* | Actinopterygies | large pelagics | 458 | 4.4 | 8.5 |
| Torpedinidae | *Torpedo marmorata* | Chondrichthyes | large demersals | 100 | 4.5 | 12.2 |
| Torpedinidae | *Torpedo nobiliana* | Chondrichthyes | large pelagics | 180 | 4.5 | 18.8 |
| Torpedinidae | *Torpedo torpedo* | Chondrichthyes | medium demersals | 60 | 4.5 | 6.2 |
| Carangidae | *Trachinotus ovatus* | Actinopterygies | medium pelagics | 70 | 3.7 | 2.5 |
| Trachinidae | *Trachinus araneus* | Actinopterygies | medium demersals | 45 | 4 |  |
| Trachinidae | *Trachinus draco* | Actinopterygies | medium demersals | 53 | 4.2 |  |
| Trachinidae | *Trachinus radiatus* | Actinopterygies | medium demersals | 50 |  |  |
| Trachipteridae | *Trachipterus trachypterus* | Actinopterygies | large pelagics | 300 | 4.5 | 7.6 |
| Carangidae | *Trachurus trachurus* | Actinopterygies | medium pelagics | 70 | 3.6 | 5.1 |
| Trichiuridae | *Trichiurus lepturus* | Actinopterygies | large pelagics | 234 | 4.4 | 1.3 |
| Triglidae | *Trigla lyra* | Actinopterygies | medium demersals | 60 | 3.5 | 5.8 |
| Tripterygiidae | *Tripterygion delaisi* | Actinopterygies |  | 8.9 | 3.4 | 0.9 |
| Gadidae | *Trisopterus luscus* | Actinopterygies | medium demersals | 46 | 3.7 | 3.3 |
| Gadidae | *Trisopterus minutus* | Actinopterygies | medium demersals | 40 | 3.8 | 4 |
| Sciaenidae | *Umbrina cirrosa* | Actinopterygies |  | 73 | 3.5 | 2.3 |
| Uranoscopidae | *Uranoscopus scaber* | Actinopterygies | medium demersals | 40 | 4.4 | 2.7 |
| Xiphiidae | *Xiphias gladius* | Actinopterygies | large pelagics | 455 | 4.5 | 3.1 |
| Labridae | *Xyrichtys novacula* | Actinopterygies |  | 38 | 3.1 | 2.6 |
| Gobiidae | *Zebrus zebrus* | Actinopterygies |  | 5.5 |  | 0.9 |
| Scophthalmidae | *Zeugopterus regius* | Actinopterygies | small demersals | 20 | 3.4 | 1.4 |
| Zeidae | *Zeus faber* | Actinopterygies | medium demersals | 90 | 4.5 | 2.2 |
| Gobiidae | *Zosterisessor ophiocephalus* | Actinopterygies |  | 25 | 3.1 | 2 |
| Trachipteridae | *Zu cristatus* | Actinopterygies | large pelagics | 118 | 4.5 | 4.3 |

1. Maximum body length. [↑](#footnote-ref-2)
2. Trophic level. [↑](#footnote-ref-3)
