# Supplementary material for: Coding Early Naturalists' Accounts into Long-Term Fish Community Changes in the Adriatic Sea (1800–2000)
Source: PLoS One. 2010 Nov 17;5(11):e15502. doi: 10.1371/journal.pone.0015502 (PMC2984504; doi:10.1371/journal.pone.0015502)
Supplement: Table S7 — Categories of taxon-specific properties based on information from Fishbase. (DOC) [file pone.0015502.s009.doc]

Table S7. Categories of *taxon*-specific properties based on information from Fishbase.

| *Taxon*-specific properties | |
| --- | --- |
| Taxonomic group | Actinopterygies |
|  | Chondrichthyes |
| Functional group | small demersals (L_max_ ≤ 30 cm) |
|  | medium demersals (30 cm < L_max_ ≤ 90 cm) |
|  | large demersals (L_max_ > 90 cm) |
|  | small pelagics (L_max_ ≤ 30 cm) |
|  | medium pelagics (30 cm < L_max_ ≤ 90 cm) |
|  | large pelagics (L_max_ > 90 cm) |
| Maximum body length (cm) | L_max_ ≤ 25 |
|  | 25 < L_max_ ≤ 55 |
|  | 55 < L_max ≤_ 120 |
|  | 120 < L_max ≤_ 250 |
|  | L_max_ > 250 |
| Age at sexual maturity (years) | Age ≤ 1 |
|  | 1 < Age ≤ 2 |
|  | 2 < Age ≤ 4 |
|  | 4 < Age ≤ 6 |
|  | 6 < Age ≤ 8 |
|  | 8 < Age ≤ 10 |
|  | 10 < Age ≤ 25 |
