# Supplementary material for: Coding Early Naturalists' Accounts into Long-Term Fish Community Changes in the Adriatic Sea (1800–2000)
Source: PLoS One. 2010 Nov 17;5(11):e15502. doi: 10.1371/journal.pone.0015502 (PMC2984504; doi:10.1371/journal.pone.0015502)
Supplement: Table S8 — Results of the analysis of trends for fish community structure indicators (N = 255 species; years 1800–1950), where significant (α = 0.1) slopes are shown in bold. (DOC) [file pone.0015502.s010.doc]

Table S8. Results of the analysis of trends for fish community structure indicators (N = 255 species; years 1800–1950), where significant (α = 0.1) slopes are shown in bold.

| Fish community structure indicators | | β | r^2^ | F | p |
| --- | --- | --- | --- | --- | --- |
|  | mean Trophic level | -0.016 | 0.211 | 2.339 | 0.201 |
| Proportion in the fish community of | **Chondrichthyes** | **-1.692** | **0.627** | **6.734** | **0.060** |
| Proportion in the fish community of | small demersals | 3.170 | 0.411 | 2.791 | 0.170 |
|  | medium demersals | 1.461 | 0.270 | 1.483 | 0.290 |
|  | **large demersals** | **-1.671** | **0.577** | **5.466** | **0.079** |
|  | small pelagics | -2.546 | 0.261 | 1.415 | 0.300 |
|  | medium pelagics | -0.410 | 0.045 | 0.188 | 0.687 |
|  | large pelagics | 0.029 | 0.000 | 0.001 | 0.974 |
| Proportion in the fish community of species with | L_max_[[1]](#footnote-2) ≤ 25 | 0.388 | 0.055 | 0.231 | 0.656 |
|  | **25 < L_max_ ≤ 55** | **4.276** | **0.779** | **14.092** | **0.020** |
|  | 55 < L_max_ ≤ 120 | -3.979 | 0.420 | 2.903 | 0.164 |
|  | **120 < L_max_ ≤ 250** | **-1.066** | **0.650** | **7.437** | **0.053** |
|  | L_max_ > 250 | 0.359 | 0.170 | 0.822 | 0.416 |
| Proportion in the fish community of species with | **Age[[2]](#footnote-3) ≤ 1** | **2.788** | **0.799** | **15.918** | **0.016** |
|  | 1 < Age ≤ 2 | -0.571 | 0.033 | 0.137 | 0.730 |
|  | 2 < Age ≤ 4 | 0.364 | 0.032 | 0.133 | 0.734 |
|  | 4 < Age ≤ 6 | 0.364 | 0.411 | 2.796 | 0.170 |
|  | 6 < Age ≤ 8 | -1.708 | 0.266 | 1.452 | 0.294 |
|  | 8 < Age ≤ 10 | 0.404 | 0.305 | 1.754 | 0.256 |
|  | 10 < Age ≤ 25 | -0.401 | 0.059 | 0.253 | 0.641 |

1. Maximum body length (cm). [↑](#footnote-ref-2)
2. Age at sexual maturity (years). [↑](#footnote-ref-3)
