# Supplementary material for: Coding Early Naturalists' Accounts into Long-Term Fish Community Changes in the Adriatic Sea (1800–2000)
Source: PLoS One. 2010 Nov 17;5(11):e15502. doi: 10.1371/journal.pone.0015502 (PMC2984504; doi:10.1371/journal.pone.0015502)
Supplement: Table S9 — Results of the analysis of trends for fish community structure indicators (N = 87 species/groups of species; years = 1800–2000), where significant (α = 0.1) slopes are shown in bold. (DOC) [file pone.0015502.s011.doc]

Table S9. Results of the analysis of trends for fish community structure indicators (N = 87 species/groups of species; years = 1800–2000), where significant (α = 0.1) slopes are shown in bold.

| Fish community structure indicators | | β | r^2^ | F | p |
| --- | --- | --- | --- | --- | --- |
|  | mean Trophic level | -0.039 | 0.305 | 2.640 | 0.155 |
| Proportion in the fish community of | **Chondrichthyes** | **-1.664** | **0.548** | **7.264** | **0.036** |
| Proportion in the fish community of | small demersals | -0.675 | 0.034 | 0.209 | 0.664 |
|  | medium demersals | -1.077 | 0.094 | 0.622 | 0.460 |
|  | **large demersals** | **-2.898** | **0.611** | **9.414** | **0.022** |
|  | small pelagics | 5.738 | 0.343 | 3.132 | 0.127 |
|  | medium pelagics | -0.671 | 0.334 | 3.015 | 0.133 |
|  | large pelagics | -0.430 | 0.076 | 0.493 | 0.509 |
| Proportion in the fish community of species with | L_max_[[1]](#footnote-2) ≤ 25 | 4.415 | 0.385 | 3.758 | 0.101 |
|  | 25 < L_max_ ≤ 55 | 0.916 | 0.076 | 0.497 | 0.507 |
|  | **55 < L_max_ ≤ 120** | **-3.901** | **0.517** | **6.418** | **0.044** |
|  | **120 < L_max_ ≤ 250** | **-1.793** | **0.506** | **6.151** | **0.048** |
|  | L_max_ > 250 | 0.172 | 0.089 | 0.588 | 0.472 |
| Proportion in the fish community of species with | Age[[2]](#footnote-3) ≤ 1 | 0.483 | 0.101 | 0.672 | 0.444 |
|  | 1 < Age ≤ 2 | 5.099 | 0.335 | 3.020 | 0.133 |
|  | 2 < Age ≤ 4 | -2.461 | 0.362 | 3.399 | 0.115 |
|  | **4 < Age ≤ 6** | **-0.979** | **0.398** | **3.967** | **0.093** |
|  | 6 < Age ≤ 8 | -1.579 | 0.286 | 2.409 | 0.172 |
|  | 8 < Age ≤ 10 | 0.125 | 0.071 | 0.460 | 0.523 |
|  | 10 < Age ≤ 25 | -0.735 | 0.174 | 1.267 | 0.303 |

1. Maximum body length (cm). [↑](#footnote-ref-2)
2. Age at sexual maturity (years). [↑](#footnote-ref-3)
