# Supplementary material for: Coding Early Naturalists' Accounts into Long-Term Fish Community Changes in the Adriatic Sea (1800–2000)
Source: PLoS One. 2010 Nov 17;5(11):e15502. doi: 10.1371/journal.pone.0015502 (PMC2984504; doi:10.1371/journal.pone.0015502)
Supplement: Materials and Methods S1 — (DOC) [file pone.0015502.s012.doc]

**Supporting Information**

**Historical data rescue**

*Archival research*

We carried out an extensive survey in libraries and archives in Venice, Padua, Rome, Trieste, Chioggia (Italy) and Split (Croatia) from January 2007 to March 2008 to collect reports, books and scientific publications dealing with fisheries and fish fauna in the Northern Adriatic Sea (Mediterranean Sea). We examined approximately 500 documents and acquired around 300. Such documents included naturalists’ lists of species and descriptions of fish fauna, grey literature dealing with fishing activities, landing statistics and official governmental reports on fisheries and fishing fleets in the area of study. We compiled one dataset on the basis of naturalists’ descriptions of fish fauna and a second dataset on the basis of landing statistics.

*Naturalists’ descriptions of fish fauna*

The main sources of information on fish fauna in the Northern Adriatic Sea (Mediterranean Sea) between the 19^th^ century and the second half of the 20^th^ century are the historical records of Italian and Austro-Hungarian naturalists, who lived and worked in Venice, Chioggia and Trieste (in present-day Italy) and in Rijeka, Rovinj and Split (in present-day Croatia).

We compiled a dataset with information on fish species reported by 36 naturalists, covering a period of about 150 years (1818–1956) (Table S1). Invertebrates and mammals were not considered in this work. Naturalists’ knowledge of fish fauna was primarily based on direct observations at fish markets and at ports, on interviews with fishermen, on literature and on the analysis of natural history museum collections [1]. These documents contain species lists, descriptions of species’ perceived abundance and insights into the main ecological characteristics of the species.

We updated species synonymies according to the modern nomenclature by comparing old scientific names to global species databases [2] and modern taxonomy books [3-5]. In the naturalists’ books, 394 fish species were described in terms of presence/absence, perceived abundance, habitat preferences, seasonality, size, and other life-history traits. Approximately 139 fish species were excluded from the dataset. The excluded species included *i)* species cited by fewer than five authors; *ii)* species belonging to freshwater habitats; *iii)* species that were misreported (e.g., the Atlantic cod *Gadus morhua*); and *iv)* species with erroneous names and species that do not exist (e.g., *Laeviraja morula* and *Notidanus barbarus*). Exclusion of these species resulted in the description of 255 valid species.

We ranked each observation of perceived abundance using a four-level coding system (i.e., very rare, rare, common and very common). Coding was based on naturalists’ descriptions of fish species; for instance, if a species was described as “found accidentally” or “occasionally” it was coded as very rare, while a species that was cited without a description of its abundance was coded as not defined.

*Landing statistics*

The landing statistics used were from major fish markets or wide coastal areas for which landing logbooks had been established by national or local authorities. These sources (henceforth termed markets) cover the period between 1874 and 2000 (Table S2). These fish markets were historically the most important of the Northern Adriatic Sea [6-9], and fleets supplying the markets exploited large areas of this basin [10]. Annual landings were given for each species or group of species in terms of wet weight (kg/years), with a different taxonomic resolution according to different sources.

**Preliminary treatment of the raw data**

We aggregated the observations into periods of 25 years because the number of species described by each naturalist was variable and the landings data from markets were not continuous. The class of perceived abundance for each species (*i*) in each period (*p*) was assigned to the most frequent class (modal value) observed in that period.

Landings (*Y_i,j,m_*) for each species (*i*), year (*j*) and market (*m*) were used to calculate the observed relative composition ($\tilde{Y}_{i,p}$) of each species for each period. $\tilde{Y}_{i,p}$ was defined by:

(1)

where *M_p_* was the total number of markets in each period (*p*) with at least one year of data, and *Ŷ_i,p,m_* was the mean annual landing of species *i* in period *p* at market *m*:

$\hat{Y}_{i,p,m}=\frac{\sum_{j\in p} Y_{i,j,m}}{D_{i,p,m}}$ (2)

where *D_i,p,m_* was the number of years with landing data for species *i* in period *p* at market *m*.

**Intercalibration and integration of the two datasets**

*Intercalibration*

Periods with overlapping information (1876–1900, 1901–1925 and 1926–1950) were considered for intercalibration of the two datasets.

Because landing statistics often report information with coarse biological resolution (genus or family), in some cases, we aggregated species into higher taxonomic groups (Table S3). The perceived abundance of the aggregated groups was defined as the highest rank among the ranks of the aggregated species (e.g., if *Acipenser naccarii* was ranked as very common, while *Acipenser sturio* was ranked as common, thus the group *Acipenser* spp. was ranked as very common). This process led to the definition of two datasets containing qualitative and quantitative information on 98 species/groups of species for the period 1876–1950.

We followed three steps for the intercalibration of the two datasets for each period (*p*).

**Step 1:** We computed the cumulative frequency of the species (П*_k,p_*) for each naturalists’ class (*X_k_*) of perceived abundance (see Figure 2a in the Main Text):

$\text{Π}_{k,p}=\sum_{1}^{k} \text{Φ}(X_{j,p})$ (3)

where Φ*(X_j,p_)* was the frequency of the species in class *X_j_* during the period *p*; *X_1_* = very rare, *X_2_* = rare, *X_3_* = common and *X_4_* = very common.

**Step 2:** We computed the percentiles for the landings corresponding to the cumulative frequency of the species in each class of perceived abundance (П*_k,p_*). These percentiles represent the class limits (*L_k,p_*) that allow the subdivision of species into four classes of landing proportions whose cumulative frequency was the same as for step 1 (see Figure 2b in the Main Text) (Table S4):

$\int_{0}^{L_{k,p}} f(\mathop{\mathop{Y}\limits^{\sim} _{i,p})}\limits=\Pi_{k,p}$ (4)

where *f($\tilde{\text{Y}}_{\text{i,p}}$)* was the frequency distribution of species *i* in the landing proportions in the period *p*.

**Step 3:** We computed class weights (*w_k,p_*) to assign the qualitative classes of perceived abundance (*X_k_*) by taking the geometric mean of the consecutive class limits (see Figure 2b in the Main Text):

$w_{k,p}=\left[L_{k,p}\text{* }L_{k+1,p}\right]^{\frac{1}{2}}$ (5)

We applied a jack-knife resampling technique [11] to estimate sample statistics (median, mean and percentiles) by computing class limits and weights 1000 times for the three periods with overlapping information (*p*), excluding 10 randomly extracted data from the naturalists’ and landings datasets in each calculation.

*Integration*

While the naturalists’ dataset contains information for 255 species, landing statistics from the periods 1951–1975 and 1976–2000 report information for 87 species/groups of species. Thus, the integration of the 200-year-long time series (1800–2000) was completed for only this subset of species.

Different class limits resulted from intercalibration of different periods (Table S4). We used the median values of the whole set of class limit replicates (global class limits) (Table S4, last raw) to transform the landing data for the periods 1951–1975 and 1976–2000 into classes of perceived abundance. We tested the acceptability of this choice by counting how many errors were made in reconstructing species’ perceived abundance in the periods with overlapping information and statistically comparing this number to the result of random classifications. To this end, we defined a vector by labeling the cases in which the perceived abundance of a species was properly reconstructed with a value of 1 and labeling the other cases with a value of 0. We tested the null hypothesis (*H_0_*) that this vector was significantly different from a vector defined by randomly assigning the same number of 0s and 1s using the *χ^2^* test and the binomial distribution. This test showed that the reconstructed array was significantly different from a random array at *p* = 10^-6^ and that the procedure failed in 34% of cases.

Different choices of global class limits led to significant reconstruction. In fact, the application of the abovementioned test to 60000 sets of class limits, obtained by randomly choosing values within the range of the class limits computed in the intercalibration, indicated that about 1200 sets define significant (*α* = 0.05) reconstructions. This result suggested a possible range of variation for each global class limit and indicated that our choice (the median) was included in such a range of variation.

**Fish community structure indicators**

*Calculation*

A set of fish community structure indicators (*Z_p_*) was used to explore changes over time in fish assemblages. These indicators were computed for each period (*p*) as the weighted averages of time-independent *taxon*-specific properties (*z_i_*) using identified class weights (*w_k,i,p_*). We used all sets of class weights (*N* = 3000) computed using the intercalibration (Table S5).

$Z_{p}=\frac{\sum_{i} w_{k,i,p}*z_{i}}{\sum_{i} w_{k,i,p}}$ (6)

Trophic level, taxonomic group, functional group, maximum body length and age at sexual maturity [2] were used as *taxon*-specific properties (*z_i_*) (Table S6). The taxonomic group, functional group, maximum body length and age at sexual maturity were categorised as indicated in Table S7, and *z_i_* was a binary value indicating whether the species belonged to one of the categories or not (1 or 0, respectively).

We performed the analysis of temporal trends using a linear regression of the median values of the fish community structure indicators. This analysis was performed on *i)* the entire naturalists’ dataset (*N* = 255 species) for the period 1800–1950 (Table S8) and *ii)* a subset of species (*N* = 87 species/groups of species) for the period 1800–2000 (Table S9).

*Sensitivity of temporal trends*

Class weights follow a logarithmic scale (see Figure 3 in the Main Text), and in our Discussion we suggest that this finding could be useful for cases in which intercalibration is not possible. To support this speculation, we tested the sensitivity of the long-term temporal trends of fish community structure indicators to variation of the base of the logarithmic scale that defines the weights. The sensitivity was tested by analyzing changes in the regression coefficients (*β*) and their significance (*p*-value) using 250 alternative sets of class weights obtained from different logarithmic scales (Figure S1). The results demonstrate that, in spite of large variations in the sets of class weights, the sign of the temporal trends (*β*) did not change, and the effects on the statistical significance (*p*-value) of the trends were modest (Figure S2). Therefore, it is possible to conclude that our results are robust with respect to variation of the base of the logarithmic scale, supporting the use of general logarithmic scales for weighting qualitative landing data.

**References**

1. Nardo GD (1824) Osservazioni ed aggiunte all'Adriatica ittiologia pubblicata dal sig. cav. Fortunato Luigi Naccari presentate dal sig. Domenico Nardo al sig. Giuseppe Cernazai di Udine. Giornale di Fisica, Chimica e Storia Naturale, Medicina ed Arti 7: 222-234.

2. Froese R, Pauly D (2009) FishBase. World Wide Web electronic publication. Available: [www.fishbase.org](http://www.fishbase.org/). Accessed 2009 March.

3. Tortonese E (1956) Fauna d’Italia (Vol II)*.* Bologna: Calderini. 334 p.

4. Tortonese E (1970) Fauna d’Italia (Vol X)*.* Bologna: Calderini. 551 p.

5. Tortonese E (1975) Fauna d’Italia (Vol XI)*.* Bologna: Calderini. 636 p.

6. Faber GL (1883) The fisheries in the Adriatic and the fish thereof. London: Bernard Quaritch. 297 p.

7. Levi Morenos D (1916) L’emigrazione peschereccia pel lavoro nell’Adriatico. Memorie del Regio Comitato Talassografico Italiano 32: 9-14.

8. D’Ancona U (1926) Dell’influenza della stasi peschereccia del periodo 1914-18 sul patrimonio ittico dell’Alto Adriatico. Memorie del Regio Comitato Talassografico Italiano 126: 5-91.

9. D’Ancona U (1949) Rilievi statistici sulla pesca nell’Alto Adriatico. Atti dell’Istituto Veneto di Scienze, Lettere ed Arti 108: 41-53.

10. Botter L, Giovanardi O, Raicevich S (2006) The migration of the Chioggia fishing-fleet in the Adriatic Sea between the 19^th^ and the early 20^th^ centuries. J Mediterr Stud 16(1/2): 27-44.

11. Crowley PH (1992) Resampling methods for computation-intensive data analysis in ecology and evolution. Annu Rev Ecol Syst 23: 405-447.

**Figure legends**

Figure S1. Sets of class weights (N = 250) used to test the sensitivity of temporal trends of fish community structure indicators to the logarithmic base. The set of class weights derived from the intercalibration (estimated base = 35.1) is represented by white squares.

Figure S2. Graphs showing how *β* and the *P*-value of temporal trends of fish community structure indicators vary when using different sets of class weights. (a) large demersals; (b) Chondrichthyes; (c) species that reach sexual maturity between the 4^th^ and 6^th^ years of life; (d) species with a maximum body length between 120 and 250 cm.
